# Activating the Osteoblastic USP26 Pathway Alleviates Multi‐Organ Fibrosis by Decreasing Insulin Resistance

**DOI:** 10.1002/advs.202512424

**Published:** 2025-12-19

**Authors:** Jiyuan Tang, Wenkai Ye, Liang He, Zhou Dan, Leilei Chang, Zijie You, Yuanyue Jiang, Guoqing Tang, Lianfu Deng, Changwei Li

**Affiliations:** ^1^ Department of Orthopedics Shanghai Key Laboratory for Prevention and Treatment of Bone and Joint Diseases Shanghai Institute of Traumatology and Orthopedics Ruijin Hospital Shanghai Jiao Tong University School of Medicine Shanghai China; ^2^ Department of Orthopedics Kunshan Hospital of Chinese Medicine Affiliated Hospital of Yangzhou University Suzhou Jiangsu China; ^3^ Institute of Traumatology and Orthopedics Kunshan Hospital of Chinese Medicine Affiliated Hospital of Yangzhou University Suzhou Jiangsu China

**Keywords:** FSTL1, insulin resistance, multiorgan fibrosis, osteoblast, USP26

## Abstract

Osteoblast dysfunction contributes to systemic metabolic disorders by inducing insulin resistance (IR), a key factor in metabolic‐related fibrosis. Therefore, the axis of osteoblast dysfunction, IR, and multi‐organ fibrosis represents a crucial pathological pathway. This study revealed that the deletion of Ubiquitin Specific Peptidase 26 (USP26) in osteoblasts leads to decreased bone formation along with multi‐organ fibrosis associated with IR. Mechanistically, the loss of USP26 decreases histone H3 lysine 18 lactylation (H3K18LA) in the promoter region of KH‐Type Splicing Regulatory Protein (KSRP), resulting in decreased expression of KSRP and decreased alternative splicing of follistatin‐like protein 1 (FSTL1) mRNA by KSRP. Elevated FSTL1 expression causes IR and high blood glucose levels, which leads to advanced glycation end‐product (AGE) accumulation in the blood and multi‐organ fibrosis. Activation of the USP26 pathway, specifically in osteoblasts, through extracellular vesicle‐based bone‐targeting drugs or mechanical loading can effectively prevent multi‐organ fibrosis induced by IR. This study uncovered a causal relationship between skeletal degeneration and metabolism‐related fibrosis, and highlights osteoblastic USP26 as a promising therapeutic target for addressing multi‐organ fibrosis associated with IR.

## Graphical abstract

The loss of Ubiquitin Specific Peptidase 26 (USP26) in osteoblasts results in decreased bone formation, as well as multi‐organ fibrosis associated with insulin resistance (IR). Mechanistically, the absence of USP26 reduces glycolysis and lactate accumulation, leading to decreased histone H3 lysine 18 lactylation (H3K18LA) in the promoter region of KH‐type splicing regulatory protein (KSRP). This results in lower transcription of KSRP and diminished alternative splicing of follistatin‐like protein 1 (FSTL1) mRNA by KSRP. Consequently, the elevated FSTL1 contributes to IR and elevated blood glucose levels. This results in the accumulation of advanced glycation end products (AGEs) in the blood, ultimately leading to multi‐organ fibrosis. Furthermore, activating the USP26 pathway specifically in osteoblasts through extracellular vesicle (EV)‐based bone‐targeting drug or compression loading can effectively prevent multi‐organ fibrosis induced by IR.

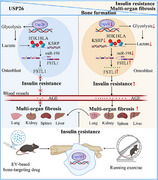

## 1 Introduction

Multiple organ fibrosis has emerged as a significant challenge in global public health, serving as the final common pathological endpoint for diverse chronic disorders [1, 2]. Its hallmark pathological feature is abnormal extracellular matrix (ECM) accumulation, which triggers the replacement of parenchymal cells with fibrous scar tissue in organs, culminating in irreversible organ failure [3, 4]. Current research has validated that chronic low‐grade inflammation [5], metabolic syndrome [6], oxidative stress damage [7], and the cellular senescence microenvironment can propel fibrosis through distinct mechanisms [8]. Insulin resistance (IR) has been identified as a central pathogenic element in metabolism‐related fibrosis [9, 10]. With the increasing prevalence of obesity and correlated metabolic ailments, the mechanisms of multi‐organ fibrosis catalyzed by IR have become a key focus area of research. Molecular analyses have shown that IR triggers myofibroblast transdifferentiation via various pathways [11]. In addition, IR can promote the pathological accumulation of ECM components, such as type I/III collagen, ultimately sparking structural reshaping of vital organs, such as the heart [12], liver [13], kidneys [14], and lungs [15]. Existing clinical interventions include thiazolidinedione medications [16, 17], metabolic surgery [18], and exercise therapy, which decelerate fibrosis progression by enhancing insulin sensitivity [19, 20, 21]. Nevertheless, unraveling the intricate pathogenic mechanisms underlying IR is indispensable for formulating strategies to reduce IR‐related fibrosis and provide a theoretical foundation for personalized treatment.

The skeletal system, now recognized as an endocrine organ [22, 23], is implicated in various systemic metabolic diseases when its secretion function is disrupted. Recent studies have shown that dysfunction of osteoblasts can contribute to systemic metabolic disorders through several mechanisms [24, 25]. Dysfunctional osteoblasts can lead to metabolic disorders by altering their secretion profile [25, 26]. This includes abnormalities in the carboxylation of osteocalcin, resulting in decreased active forms [27], disruptions in adiponectin secretion [28], and upregulation of sclerostin by inhibiting the Wnt/β‐catenin signaling pathway [29]. These factors directly interfere with insulin signaling, affecting pancreatic beta cell function and peripheral tissue sensitivity to insulin. Our previous research has shown that the activation of osteoblastic Hypoxia‐Inducible Factor 1‐alpha (HIF‐1α) not only promotes bone formation but also improves symptoms of type 1 diabetes mellitus, such as increased glucose clearance, protection of pancreatic beta cells, promotion of beta cell proliferation, and stimulation of insulin secretion [30]. Considering the significant role of IR in metabolism‐related fibrosis, the axis of osteoblast dysfunction‐IR‐multi‐organ fibrosis may represent a critical pathological pathway. This study provides a new perspective for understanding the association between organ fibrosis and metabolic diseases.

In the present study, we reveal that deletion of Ubiquitin Specific Peptidase 26 (USP26) in osteoblasts results in IR and decreased bone formation, leading to multi‐organ fibrosis. Activation of the USP26 pathway, specifically in osteoblasts, through extracellular vesicle (EV)‐based bone‐targeting drugs or compression loading can effectively prevent multi‐organ fibrosis induced by IR. Thus, this study highlights osteoblastic USP26 as a promising therapeutic target for treating multi‐organ fibrosis associated with IR.

## 2 Results

### 2.1 Knockout of USP26 in Osteoblasts Impairs Osteogenic Capacity and Leads to Multi‐Organ Fibrosis

We have previously reported that USP26 facilitates osteogenic differentiation of bone marrow mesenchymal stem cells (BMSCs) and that USP26 deletion in BMSCs leads to decreased bone formation [31, 32]. Our present results show increased USP26 expression during the differentiation of BMSC and osteoblast progenitors into mature osteoblasts (Figure S1A–C), indicating that USP26 may play a vital role in osteoblast function. To further investigate the function of USP26 in bone formation and related diseases, we crossed osteocalcin‐Cre (Ocn‐Cre) mice with USP26^flox/flox^ mice to generate conditional knockout mice lacking Usp26 specifically in osteoblasts, known as USP26^flox/flox^, Ocn‐Cre^+/−^ mice (referred to as Usp26 cKO mice). Usp26^flox/flox^, Ocn‐Cre^−/−^ littermates were used as wild type (WT) controls. Osteocalcin (OCN) is one of the most abundant non‐collagenous proteins in bone [33, 34, 35]. As OCN is primarily expressed in mature osteoblasts, Ocn‐Cre is widely used for conditional gene knockout in these cells [36, 37, 38]. Previously, we crossed Ocn‐Cre transgenic mice with Rosa26 mT/mG reporter mice and found that the bones of these mice contained GFP^+^ cells in the hypertrophic chondrocyte regions of the growth plate, throughout the metaphysis, and on and around the cortical and trabecular bone surfaces. However, no GFP^+^ cells have been observed in other tissues such as the heart, liver, spleen, lung, kidney, or skeletal muscle [30]. To further assess the knockout efficiency and specificity of Usp26, we examined both the protein and mRNA levels of USP26 in osteoblasts and in other organs and tissues, including the liver, spleen, lung, and kidney, in Usp26 cKO mice and their littermate controls. Compared with littermate control mice, Usp26 cKO mice exhibited a significant reduction in USP26 protein (Figure S2A,B) and mRNA expression levels (Figure S2C), specifically in osteoblasts, whereas no significant differences in Usp26 mRNA levels were observed in the liver, spleen, lungs, and kidneys (Figure S2D).

**FIGURE 1:**
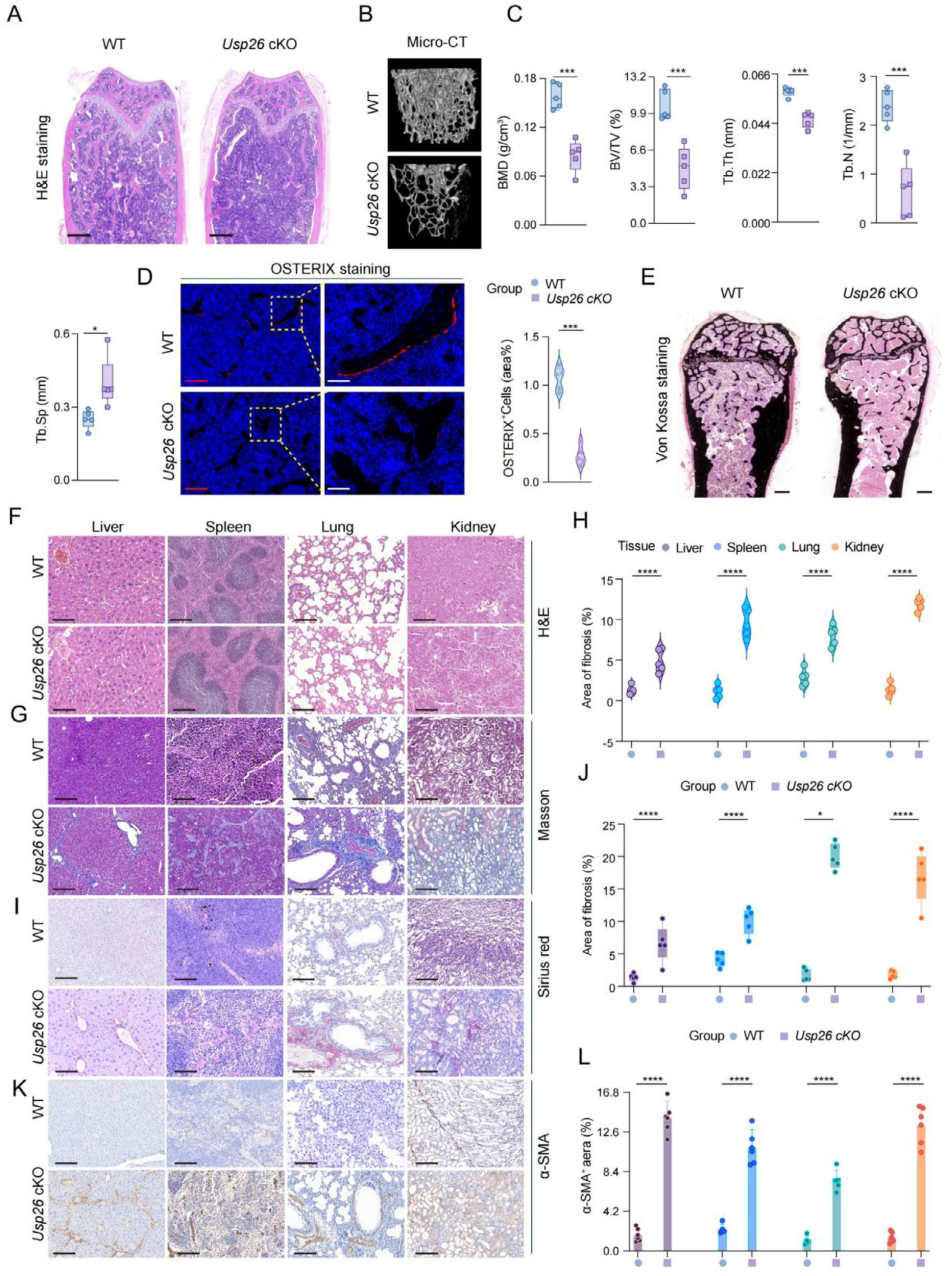
Knockout of USP26 in osteoblasts impairs osteogenic capacity and leads to multiorgan fibrosis. (A) Representative H&E staining of femoral sections from 2‐month‐old Usp26 cKO mice and their littermate controls. *n* = 5 in each group. Scale bars: 500 µm. (B) Representative micro‐CT images of femurs from 2‐month‐old Usp26 cKO mice and their littermate controls. *n *= 5 in each group. (C) Quantitative analysis of trabecular bone parameters, including BMD, BV/TV, Tb. N, Tb.Th, and Tb.Sp in 2‐month‐old Usp26 cKO mice and their littermate controls. *n *= 5 in each group. (D) OSTERIX immunofluorescence staining of femoral sections from 2‐month‐old Usp26 cKO mice and their littermate controls. *n *= 5 in each group. Red scale bars: 200 µm; white scale bars: 30 µm. (E) Representative Von Kossa staining of femoral sections from 2‐month‐old Usp26 cKO mice and their littermate controls. *n *= 5 in each group. Scale bars: 500 µm. (F) Representative H&E staining of liver, spleen, lung, and kidney sections from 24‐week‐old Usp26 cKO mice and their littermate controls. *n *= 5 in each group. Scale bars: 100 µm. (G) Representative Masson's trichrome staining of liver, spleen, lung, and kidney sections from 24‐week‐old Usp26 cKO mice and their littermate controls. *n *= 5 in each group. Scale bars: 100 µm. (H) Masson staining to count the area of fibrosis. *n *= 5 in each group. (I) Sirius red staining of liver, spleen, lung, and kidney sections of 24‐week‐old Usp26 cKO mice and their littermate controls. *n *= 5 in each group. Scale bars: 100 µm. (J) Statistical fibrosis area of Sirius red staining. *n *= 5 in each group. (K) Immunohistochemical staining of liver, spleen, lung, and kidney sections α‐SMA from 24‐week‐old Usp26 cKO mice and their littermate controls. *n *= 5 in each group. Scale bars: 100 µm. (L) Statistical analysis of α‐SMA. *n *= 5 in each group. Data are presented as mean ± SEM. Statistical significance was determined by a two‐tailed Student's *t*‐test. *p < 0.05, ***p < 0.001, ****p < 0.0001.

Hematoxylin and eosin (H&E) staining revealed that the amount of trabecular bone was significantly lower in the femurs of 2‐month‐old Usp26 cKO mice than in those of littermate controls (Figure 1A). Further analysis of the trabecular bone from the distal femur metaphysis using micro‐quantitative computed tomography showed decreased cancellous bone volume/tissue volume (BV/TV), bone mineralization density, trabecular number (Tb. N), and trabecular thickness (Tb. Th), and increased trabecular separation (Tb. Sp) in the Usp26 cKO mice (Figure 1B,C). Additionally, there were fewer OSTERIX^+^ osteoblastic cells on the trabecular bone surface, indicating impaired osteogenic activity in USP26 cKO mice (Figure 1D). Von Kossa staining further demonstrated decreased mineralization levels in Usp26 cKO mice compared to littermate controls (Figure 1E).

To further investigate whether USP26 influences the osteogenic differentiation of osteoblasts in vitro, osteoblasts isolated from Usp26 cKO mice and their WT littermate controls were cultured in osteogenic medium for 0, 4, 8, and 12 days. During osteogenic differentiation, the mRNA expression levels of osteogenic markers, including alkaline phosphatase (Alp), bone morphogenetic protein‐2 (Bmp2), runt‐related transcription factor 2 (Runx2), osteocalcin (Ocn), and osterix, gradually increased. However, the expression of these genes was substantially reduced in the absence of Usp26 (Figure S1D). Consistently, the upregulated protein levels of RUNX2, OSTERIX, and ALP were also significantly impaired in the absence of USP26 during osteogenic differentiation of osteoblasts (Figure S1E,F). Additionally, ALP and alizarin red S staining showed that ALP activity and ECM mineralization were markedly reduced in Usp26 cKO osteoblasts (Figure S1G).

Abnormal functioning of osteoblasts often results in the dysfunction of peripheral organs. In this study, we investigated the effects of Usp26 deletion in osteoblasts on peripheral organs, such as the liver, spleen, lung, and kidney. Histological staining using H&E, Masson's, Sirius Red, and alpha smooth muscle actin (α‐SMA) revealed fibrosis in multiple organs. These findings collectively suggest that Usp26 deletion in osteoblasts hinders osteogenic differentiation and bone formation and leads to fibrosis in peripheral organs, including the liver, spleen, lungs, and kidneys (Figure 1F–L).

### 2.2 USP26 Deletion in Osteoblasts Leads to Multi‐Organ Fibrosis due to Insulin Resistance

**FIGURE 2:**
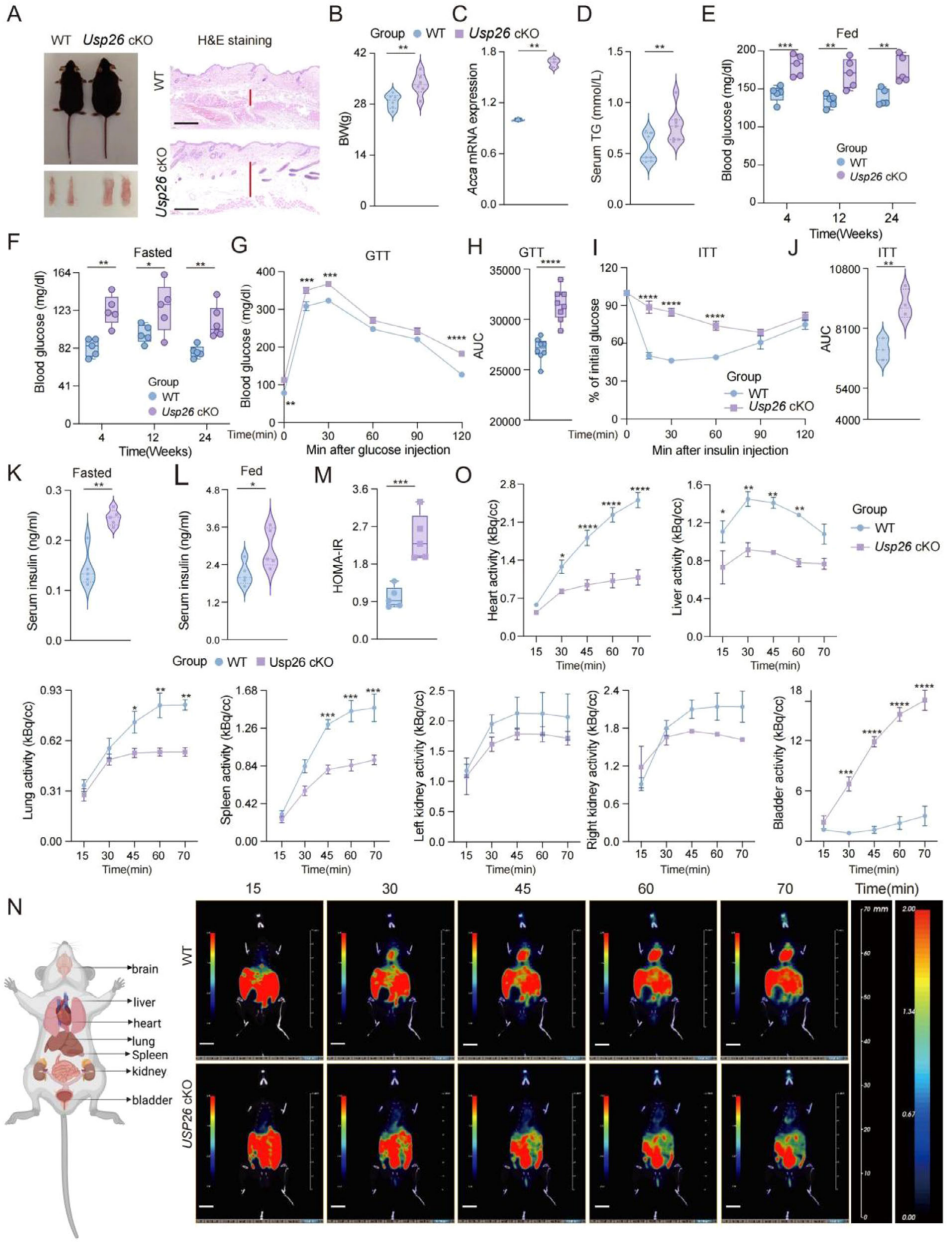
USP26 deletion in osteoblasts leads to IR. (A) Photographs of 24‐week‐old Usp26 cKO mice and their littermate controls, along with representative images of epididymal fat pads, *n *= 5 in each group; representative H&E staining of mouse abdominal skin sections. The red line indicates the thickness of the subcutaneous fat layer. *n *= 5 in each group. Black scale bars: 200 µm. (B) Body weight measurements of 24‐week‐old Usp26 cKO mice and their littermate controls. *n* = 6 in each group. (C) qPCR analysis of Acca mRNA levels in the liver of 24‐week‐old Usp26 cKO mice and their littermate controls. *n *= 3 in each group. (D) Serum triglyceride (TG) levels in 24‐week‐old Usp26 cKO mice and their littermate controls. *n* = 7 in each group. (E) Random‐fed blood glucose levels in 24‐week‐old Usp26 cKO mice and their littermate controls. *n *= 5 in each group. (F) Fasting blood glucose levels in 24‐week‐old Usp26 cKO mice and their littermate controls. *n *= 5 in each group. (G, H) GTT and corresponding area under the curve (AUC) for 24‐week‐old Usp26 cKO mice and their littermate controls. *n *= 6 in each group. (I, J) ITT and corresponding AUC for 24‐week‐old Usp26 cKO mice and their littermate controls. *n *= 6 in each group. (K)Fasted serum insulin levels in 24‐week‐old Usp26 cko mice and their littermate controls. *n *= 5 in each group. (L) Fed serum insulin levels in 24‐week‐old Usp26 cko mice and their littermate controls. *n *= 5 in each group. (M) HOMA values levels in 24‐week‐old Usp26 cKO mice and their littermate controls. *n *= 5 in each group. (N) Representative time‐lapse whole‐body micro‐PET imaging of ^18^F‐FDG uptake in 24‐week‐old Usp26 cKO mice and their littermate controls. *n *= 5 in each group. Scale bars: 10mm. (O) Statistical analysis of standardized uptake value (SUV) for organs based on micro‐PET results. *n *= 5 in each group. Data are presented as mean ± SEM. Statistical significance was determined by two‐tailed Student's *t*‐test in (B), (C), (D), (H), (J), (K), (L), and (M), two‐way ANOVA in (E), (F), (G), (I), and (O). *p < 0.05, **p < 0.01, ***p < 0.001, ****p < 0.0001.

Persistent inflammation [39] and metabolic syndrome [40] are important factors that drive fibrotic progression. In this study, we found no significant changes in the number of myeloid cells, macrophages, or T cells in the blood of Usp26 cKO mice (Figure S3A–C). However, we observed that during daily maintenance, Usp26 cKO mice were more obese, with a significant increase in body weight and epididymal and subcutaneous fat compared to their littermate controls (Figure 2A, B). Furthermore, after knocking out USP26 in osteoblasts, we found that the gene Acca, which is involved in fat synthesis in the liver, and serum triglyceride levels were significantly elevated (Figure 2C,D). Additionally, blood glucose measurements revealed that both male and female Usp26 cKO mice had higher blood glucose levels at 4, 12, and 24 weeks under ad libitum (Figure 2E) and fasting conditions (Figure 2F). These findings suggest that the absence of Usp26 in osteoblasts leads to a physiological metabolic syndrome and hyperglycemia in mice, which could potentially cause fibrosis in knockout mice.

IR is the most critical factor in metabolic syndrome and hyperglycemia [41]. Furthermore, glucose tolerance tests (GTT) and insulin tolerance tests (ITT) conducted on Usp26 cKO and littermate control mice showed that the loss of Usp26 in osteoblasts severely impaired glucose and insulin tolerance in mice, leading to significant increases in blood glucose levels and obesity (Figure 2G–J). Additionally, Usp26 cKO mice exhibited significantly higher serum insulin levels under both fasting and random feeding conditions (Figure 2K,L). The insulin resistance index calculated using the Homeostatic Model Assessment of Insulin Resistance (HOMA‐IR) revealed that Usp26 cKO mice exhibited significant IR (Figure 2M). Furthermore, dynamic changes in glucose levels in the mice, analyzed by positron emission tomography‐computed tomography (PET‐CT), revealed that because of IR, glucose was unable to enter peripheral organs such as the heart, liver, lung, spleen, and bladder for utilization and instead accumulated in the urine of USP26 cKO mice (Figure 2N,O). These results collectively demonstrate that the deletion of USP26 in osteoblasts leads to IR, resulting in metabolic syndrome and hyperglycemia.

**FIGURE 3:**
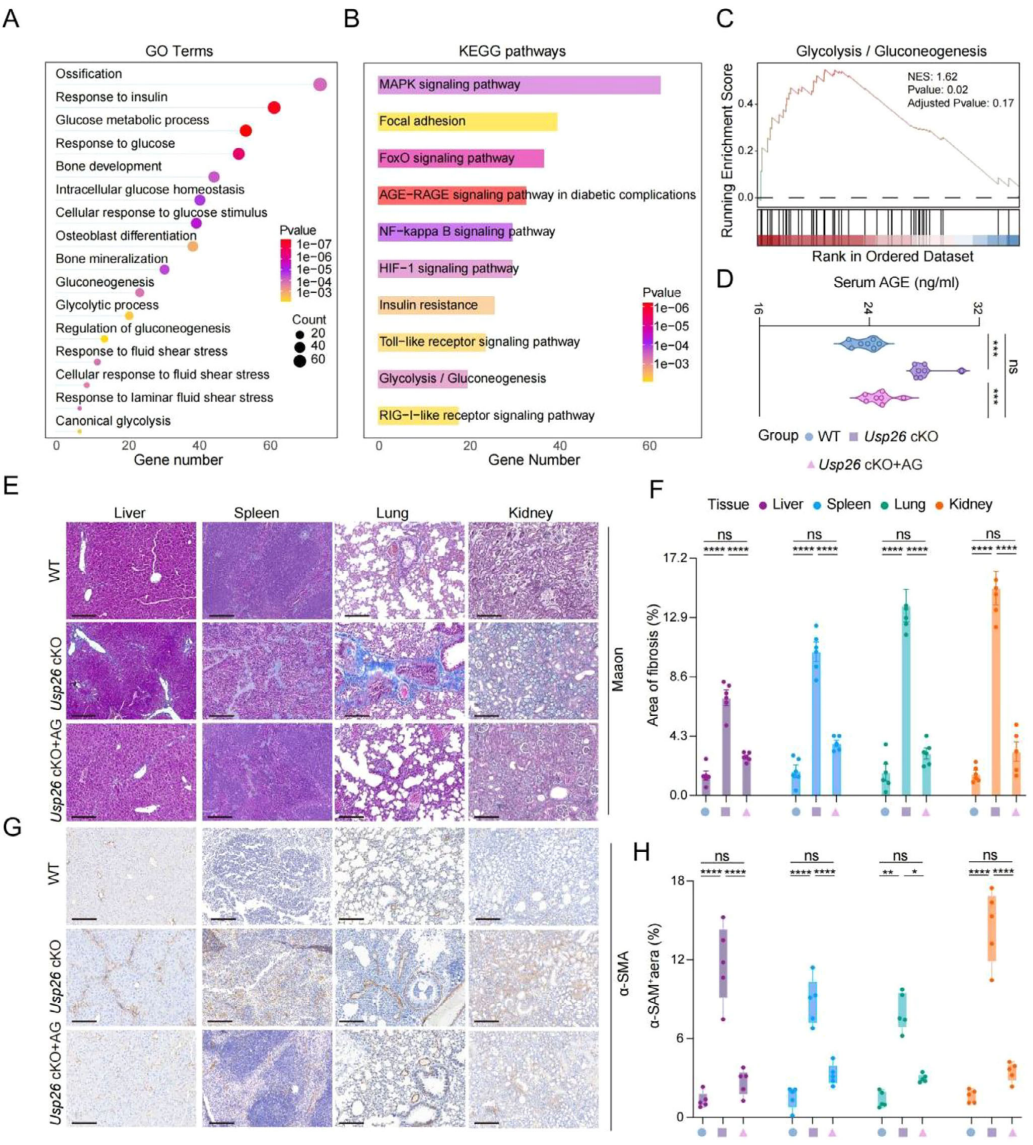
The increased AGE production was responsible for the multi‐organ fibrosis observed in Usp26 cKO mice. (A–C) Performing GO (A), KEGG (B), and GSEA (C) analysis of differentially expressed genes in Usp26 KO osteoblasts and their littermate controls. (D) Serum AGE levels in 24‐week‐old Usp26 cKO mice, their littermate controls, and Usp26 cKO mice injected with AG. *n *= 5 in each group. (E) Representative Masson's trichrome stain of liver, spleen, lung, and kidney sections from 24‐week‐old Usp26 cKO mice, their littermate controls, and Usp26 cKO mice injected with AG. *n* = 5 in each group. Scale bars: 100 µm. (F) Quantitative analysis of Masson's trichrome stain. *n *= 5 in each group. (G) Representative α‐SMA immunohistochemical staining of liver, spleen, lung, and kidney sections from 24‐week‐old Usp26 cKO mice, their littermate controls, and Usp26 cKO mice injected with AG. *n *= 5 in each group. Scale bars: 100 µm. (H) Quantitative analysis of α‐SMA expression. *n *= 5 in each group. Data are presented as mean ± SEM. Statistical significance was determined by one‐way ANOVA. *p < 0.05, **p < 0.01, ***p < 0.01, ****p < 0.0001.

Hyperglycemia is typically accompanied by the production of a large amount of advanced glycation end products (AGEs), which are a key factor in multi‐organ fibrosis [42, 43]. Our data revealed that Usp26 cKO significantly increased blood AGE levels (Figure 3D). Additionally, gene expression analysis in the Usp26 cKO and WT control osteoblasts revealed significant enrichment of the AGE‐RAGE signaling pathway in diabetic complications, in addition to bone ossification, osteoblast differentiation, insulin resistance, and glucose metabolism pathways (Figure 3A–C). Therefore, we hypothesized that the increased blood AGE levels may lead to multi‐organ fibrosis observed in Usp26 cKO mice. To test this hypothesis, we used aminoguanidine (AG), an inhibitor of AGE formation, in subsequent experiments [44]. The results showed that AG administration led to a significant decrease in blood AGE production (Figure 3D), along with significant inhibition of fibrosis in the liver, spleen, lungs, and kidneys of Usp26 cKO mice, as demonstrated by Masson's trichrome staining and α‐SMA immunohistochemistry (Figure 3E–H). Collectively, these data indicate that the loss of USP26 in osteoblasts leads to IR, which in turn causes multi‐organ fibrosis through hyperglycemia and AGE production.

### 2.3 Deletion of USP26 in Osteoblasts Leads to Insulin Resistance Through FSTL1

**FIGURE 4:**
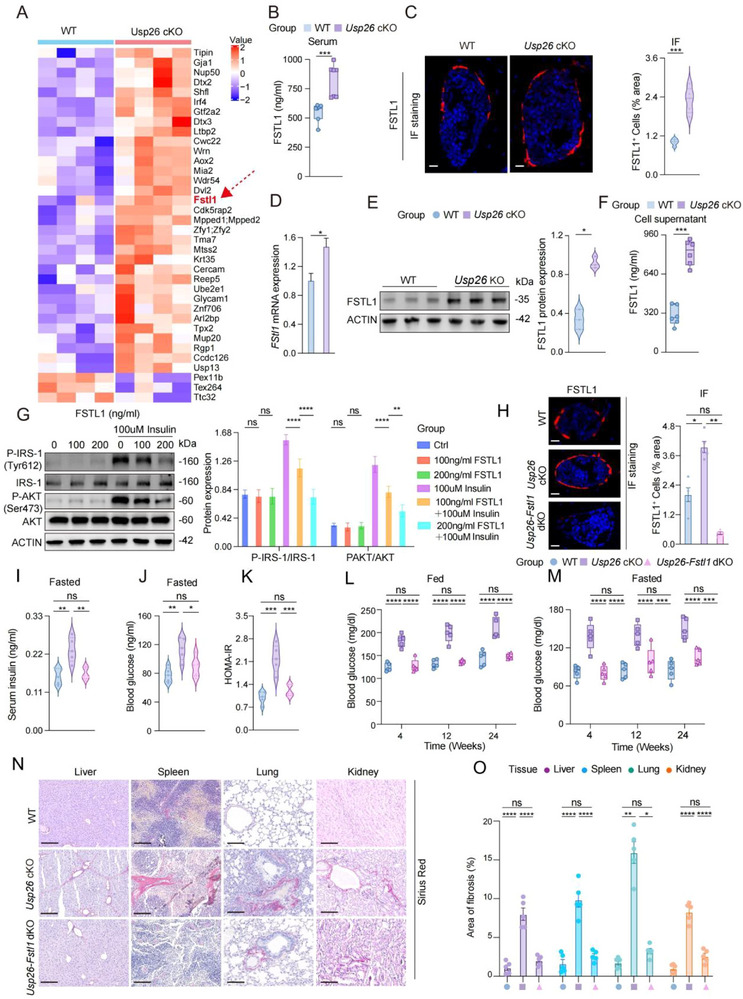
Increased secretion of FSTL1 by osteoblasts in Usp26 cKO mice promotes IR progression. (A) Proteomic analysis of bone marrow supernatant from Usp26 cKO mice and their littermate controls. (B) ELISA assay of serum FSTL1 levels in Usp26 cKO mice and their littermate controls. Controls. *n *= 5 in each group. (C) Representative immunofluorescence (IF) staining of FSTL1 in femoral sections from Usp26 cKO mice and their littermate controls, with quantitative analysis. *n *= 5 in each group. Scale bars:15µm. (D) qPCR analysis of Fstl1 mRNA levels in osteoblasts isolated from Usp26 cKO mice and their littermate controls. *n *= 3 in each group. (E) Western blot analysis of FSTL1 protein expression in osteoblasts from Usp26 cKO mice and their littermate controls, with quantification. *n *= 3 in each group. (F) ELISA assay of FSTL1 levels in conditioned medium from osteoblast cultures of Usp26 cKO mice and their littermate controls. *n *= 5 in each group. (G) Western blot analysis of phosphorylated IRS‐1 (P‐IRS‐1), total IRS‐1, AKT, and phosphorylated AKT (P‐AKT) protein levels in hepatocytes treated with varying concentrations of FSTL1 and insulin. (H) Representative FSTL1 Immunofluorescence Staining of 24‐week‐old Usp26 cKO mice, WT controls, and Usp26‐Fstl1 dKO mice. *n *= 5 in each group. Scale bars: 25 µ0m. (I, J) ELISA assay of serum insulin levels and glycemia in 24‐week‐old Usp26 cKO mice, WT controls, and Usp26‐Fstl1 dKO mice under fasting conditions. *n *= 5 in each group. (K) HOMA‐IR values. *n *= 5 in each group. (L) Random‐fed blood glucose levels in 24‐week‐old Usp26 cKO mice, WT controls, and Usp26‐Fstl1 dKO mice. *n *= 5 in each group. (M) Fasting blood glucose levels in 24‐week‐old Usp26 cKO mice, WT controls, and Usp26‐Fstl1 dKO mice. *n *= 5 in each group. (N) Sirius red staining of liver, spleen, lung, and kidney sections of 24‐week‐old Usp26 cKO mice, WT controls, and Usp26‐Fstl1 dKO mice. *n *= 5 in each group. Scale bars: 100 µm. (O) Statistical fibrosis area of Sirius red staining, *n *= 5 in each group. Data are presented as mean ± SEM. Statistical significance was determined by two‐tailed Student's *t*‐test in (B), (C), (D), (E), and (F), one‐way ANOVA in (G), (H), (I), (J), (K), and (O), two‐way ANOVA in (L)and (M). *p < 0.05, **p < 0.01, ***p < 0.001, ****p < 0.0001.

As endocrine cells, osteoblasts regulate systemic physiological activities by secreting various factors into the bone marrow cavity [45]. To clarify the mechanism by which Usp26 deletion in osteoblasts leads to insulin resistance, we first conducted a proteomic analysis to examine changes in protein expression in bone tissues. Among the 20 proteins showing the most significant differential expression, only latent transforming growth factor beta‐binding protein 2 (LTBP2), melanoma inhibitory activity 2 (MIA2), and follistatin‐like 1 (FSTL1) were identified as secreted proteins (Figure 4A). Given that FSTL1 was the only protein that demonstrated a dual association with hyperglycemia and fibrosis, we selected FSTL1 for further analysis. Enzyme‐linked immunosorbent assay results showed that Usp26 cKO led to increased serum FSTL1 levels (Figure 4B), while immunostaining confirmed that Usp26 knockout promoted FSTL1 expression in osteoblasts (Figure 4C). To determine whether Usp26 cKO led to increased FSTL1 expression and secretion in vitro, osteoblasts were isolated from USP26 cKO mice and their WT littermates. RNA and protein analyses showed that Usp26 knockout in osteoblasts enhanced FSTL1 expression (Figure 4D, E), and Usp26 cKO osteoblasts secreted substantially greater amounts of FSTL1 into the culture supernatant than controls (Figure 4F).

FSTL1 is a secreted glycoprotein and a potential mediator of IR in obesity [46]. Consistent with previous reports, we confirmed that FSTL1 downregulates the expression of insulin receptor‐related signaling pathways in response to insulin stimulation, such as the phosphorylation of insulin receptor substrate‐1 (IRS‐1) at Tyr612 and protein kinase B (AKT) at Ser473 (Figure 4G). Serine phosphorylation of IRS1 has been reported to block insulin signaling and induce IR [47, 48], FSTL1 induces a dose‐dependent phosphorylation of JNK at Thr183/Tyr185 and IRS1 at Ser307, either alone or in combination with insulin (Figure S4A,B).

To determine whether the upregulation of FSTL1 was responsible for the observed IR in Usp26 cKO mice, we crossed Usp26 cKO mice and Fstl1^flox/flox^ mice to generate mice with Usp26 and Fstl1 double knockout in osteoblasts, referred to as Usp26^flox/flox^‐Fstl1^flox/flox^‐Ocn‐Cre^+/−^ mice (later referred to as Usp26‐Fstl1 dKO mice). For the control group, Usp26^flox/flox^‐Fstl1^flox/flox^‐Ocn‐Cre^−/−^ littermates were used, referred to as Usp26‐Fstl1^flox/flox^ mice (later referred to as dKO control mice). The knockout efficiency was confirmed by FSTL1 immunostaining (Figure 4H) and analysis of Usp26 and Fstl1 mRNA expression in osteoblasts (Figure S5). Usp26‐Fstl1 dKO mice were viable and born at the expected Mendelian ratio, and their body size and weight were comparable to those of the dKO control mice.

With the downregulation of FSTL1 expression, IR calculated using fasting blood glucose and fasting insulin concentration, as indicated by HOMA‐IR (Figure 4I–K); hyperglycemia, as shown by serum glucose concentration (Figure 4L,M); and fibrosis in the liver, spleen, lungs, and kidneys, as demonstrated by Sirius Red staining (Figure 4N,O), were significantly improved in Usp26‐Fstl1 dKO mice compared to Usp26 cKO mice. FSTL1‐neutralizing antibodies are widely used in research on lung [49], liver [50, 51, 52], and dermal fibrosis [53]. To directly implicate secreted FSTL1 as a key effector in IR and multi‐organ fibrosis, we used an FSTL1‐neutralizing antibody to inhibit the function of serum FSTL1 in mice. As shown in Figure S6, treatment with FSTL1‐neutralizing antibody effectively reduced fasting blood glucose levels (Figure S6A), insulin concentrations (Figure S6B), HOMA‐IR (Figure S6C), and fibrosis in the liver, spleen, lungs, and kidneys (Figure S6D,E). These findings suggest that the deletion of USP26 in osteoblasts leads to insulin resistance and fibrosis in multiple organs through FSTL1.

### 2.4 Patients With Osteoporosis in the Diabetic Population Have Decreased Skeletal USP26 and Increased Blood Levels of FSTL1

After discovering that the loss of USP26 in osteoblasts reduces bone formation and leads to IR, hyperglycemia, and higher blood AGE levels owing to the excessive release of FSTL1 from osteoblasts in mice, we aimed to investigate whether there is a negative correlation between skeletal USP26 expression and serum levels of FSTL1 in humans.

**FIGURE 5:**
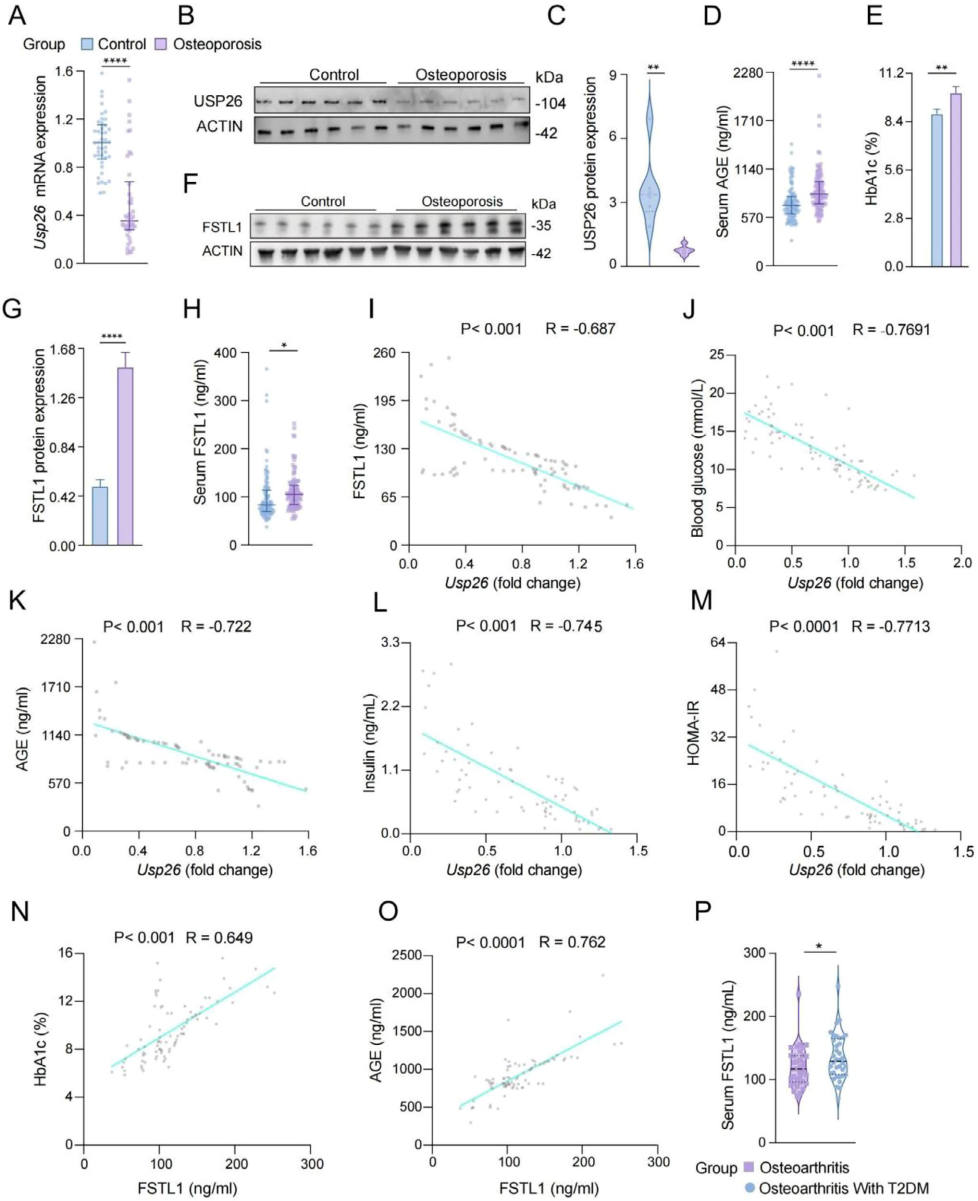
Patients with osteoporosis in the diabetic population have decreased skeletal USP26 and increased blood levels of FSTL1. (A) qPCR analysis of Usp26 mRNA in the femur of osteoporotic and non‐osteoporotic patients. *n *= 42 in each group. (B) Western blot analysis of USP26 protein expression in the femur of osteoporotic and non‐osteoporotic control patients. *n *= 6 in each group. (C) Quantitative analysis of Western blot results in (B). *n *= 6 in each group. (D) Serum concentrations of AGEs in osteoporosis patients versus control subjects. *n *= 103 in each group. (E) Serum concentrations of HbAIc in osteoporosis patients versus control subjects. *n *= 42 in each group. (F) Western blot analysis of FSTL1 protein expression in femoral tissues from osteoporotic patients and non‐osteoporotic controls. *n *= 6 in each group. (G) Quantitative analysis of Western blot results in (F). *n *= 6 in each group. (H) Serum concentrations of FSTL1in osteoporosis patients versus control subjects. *n *= 103 in each group. (I–K) Correlation analysis was conducted to study the relationship between FSTL1, blood glucose, and blood AGE levels with USP26 expression in femoral bone tissues. *n *= 84. (L, M) Correlation analysis was conducted to study the relationship between fasting insulin levels and HOMA‐IR with USP26 expression in femoral bone tissues. *n *= 68. (N, O) Correlation analysis between the levels of HbA1c and blood AGE levels with serum FSTL1 in humans. *n* = 84. (P) Analysis of plasma FSTL1 concentration in patients with osteoarthritis with or without type 2 diabetes. *n* = 36 in each group. Data are presented as mean ± SEM. Statistical significance was determined by a two‐tailed Student's *t*‐test. *p < 0.05, **p < 0.01, ****p < 0.0001.

A total of two hundred and six blood and 84 femoral tissue samples were collected from diabetic patients who underwent total knee arthroplasty for end‐stage knee osteoarthritis. Of these samples, 103 blood and 42 femoral tissue samples were obtained from patients with osteoporosis (T≤−2.5), while the remaining 103 blood and 42 femoral tissue samples were collected from patients with normal bone mass (T>−2.5). Both mRNA and protein analyses showed that USP26 expression was significantly decreased in patients with osteoporosis (Figure 5A–C). Conversely, the serum concentrations of AGE (Figure 5D) and glycated hemoglobin A1c (HbA1c) (Figure 5E), as well as both skeletal and serum FSTL1 (Figure 5F–H), were significantly elevated in these patients. Further correlation analyses revealed a negative association between skeletal USP26 expression and FSTL1 (Figure 5I), blood glucose (Figure 5J), blood AGE (Figure 5K), fasting insulin (Figure 5L), and HOMA‐IR (Figure 5M). In addition, FSTL1 was positively correlated with both HbA1c (Figure 5N) and AGE (Figure 5O).

Elevated FSTL1 levels may be partly attributable to osteoarthritis. Therefore, to further clarify the specific impact of diabetes on circulating FSTL1 levels, we included a control group of patients with osteoarthritis but without type 2 diabetes. Patients with both diabetes and osteoarthritis had significantly higher blood FSTL1 levels than those with osteoarthritis alone (Figure 5P). Collectively, these findings reveal that patients with osteoporosis have decreased skeletal USP26 and increased blood levels of FSTL1.

### 2.5 Reduced H3K18LA/KSRP Reduces the Alternative Splicing of FSTL1 mRNA

**FIGURE 6:**
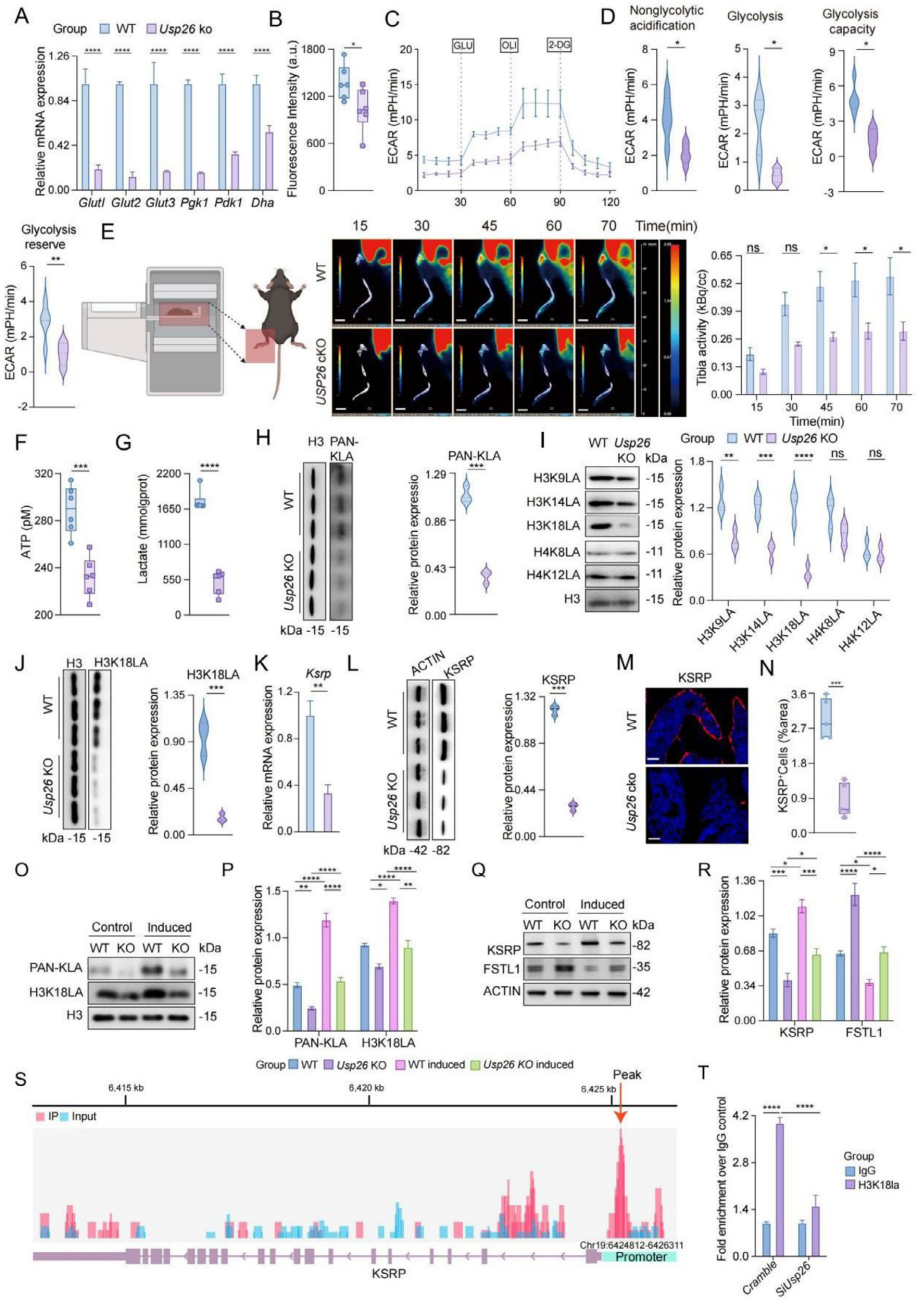
Reduced H3K18LA/KSRP reduces the transcriptional expression of FSTL1. (A) qPCR analysis of glycolysis‐related gene mRNA in osteoblasts of Usp26 cKO mice and their littermate controls. *n *= 3 in each group. (B) Fluorescent glucose uptake in osteoblasts of Usp26 cKO mice and their littermate controls. *n *= 3 in each group. (C, D) ECAR analysis of osteoblasts from Usp26 cKO mice and their littermate controls. *n *= 5 in each group. (E) Representative serial micro‐PET imaging of ^18^F‐FDG uptake in the femur (tibia) of 24‐week‐old Usp26 cKO mice and their littermate controls. *n *= 5 in each group. Scale bars: 5mm. (F) ATP concentration in osteoblasts of Usp26 cKO mice and their littermate controls. *n *= 3 in each group. (G) Lactate concentration in osteoblasts of Usp26 cKO mice and their littermate controls. *n *= 5 in each group. (H) Western blot and quantitative analysis of PAN‐KLA protein expression in osteoblasts of Usp26 cKO mice and their littermate controls.*n *= 3 in each group. (I)Western blot and quantitative analysis of H3K9LA, H3K14LA, H3K18LA, H4K8LA, and H4K12LA protein expression in osteoblasts of Usp26 cKO mice and their littermate controls. *n *= 3 in each group. (J) Western blot and quantitative analysis of H3K18LA protein expression in osteoblasts of Usp26 cKO mice and their littermate controls. *n *= 3 in each group. (K) qPCR analysis of Ksrp mRNA in osteoblasts of Usp26 cKO mice and their littermate controls. *n *= 3 in each group. (L) Western blot and quantitative analysis of KSRP protein expression in osteoblasts of Usp26 cKO mice and their littermate controls. *n *= 3 in each group. (M, N) KSRP Immunofluorescence staining and quantitative analysis in femurs of Usp26 cKO mice and their littermate controls. *n *= 5 in each group. Scale bars: 25µm. (O, P) Western blot and quantitative analysis of PAN‐KLA and H3K18LA protein expression in osteoblasts of Usp26 cKO mice and their littermate controls treated with PBS or lactate. *n *= 3 in each group. (Q, R) Western blot and quantitative analysis of KSRP and FSTL1 protein expression in osteoblasts of Usp26 cKO mice and their littermate controls treated with PBS or lactate. (S) chip‐qPCR. (T) chip‐seq. Data are presented as mean ± SEM. Statistical significance was analyzed using two‐tailed Student's *t*‐test in (A), (B), (D), (F), (G), (H), (I), (J), (K), (L) and (N), one‐way ANOVA in (P) and (R), and two‐way ANOVA in (E) and (T). *p < 0.05, **p < 0.01, ***p < 0.001, ****p < 0.0001.

We aimed to elucidate the underlying mechanisms by which USP26 regulates FSTL1 expression in osteoblasts. Gene Ontology and Kyoto Encyclopedia of Genes and Genomes enrichment, along with Gene Set Enrichment Analysis, indicated that glucose metabolic processes and glycolysis were significantly associated with differential gene expression in osteoblasts following Usp26 deletion. Correspondingly, the expression of genes related to glucose uptake and glycolysis, such as Glut1, Glut2, Glut3, Pgk1, Pdk1, and Dha, was notably reduced in Usp26^−/−^ osteoblasts (Figure 6A). Moreover, compromised glucose uptake and glycolysis in Usp26^−/−^ osteoblasts were corroborated by a fluorescent glucose uptake assay using 2‐deoxy‐D‐glucose (Figure 6B) and extracellular acidification rate (ECAR) results (Figure 6C,D). Furthermore, PET‐CT analysis of glucose uptake in the tibia/femur of cKO and WT mice indicated a significant decrease in glucose uptake in the femur and tibia of Usp26 cKO mice compared to that in WT mice (Figure 6E). Owing to the decreased glucose uptake and glycolysis, decreased intracellular lactate, ATP concentrations (Figure 6F,G), and p‐AMPK/AMPK ratios (Figure S7), were observed in Usp26^−/−^ osteoblasts. Collectively, these data demonstrate that loss of Usp26 leads to impaired glycolysis in osteoblasts.

A decrease in glycolysis and lactic acid content dampens the lactylation of histones and inhibits the transcriptional expression of target genes [54]. Therefore, we aimed to determine whether increased Fstl1 expression in Usp26^−/−^ osteoblasts was due to changes in histone lactylation. Initially, we observed significant downregulation of total protein lactylation after Usp26 knockout (Figure 6H). To identify specific histones and sites undergoing lactylation, we systematically screened common histones and their corresponding lactylation sites. Our findings revealed a significant decrease in histone H3 levels in Usp26‐deficient cells, with the most pronounced decline observed in lysine 18 lactylation of histone H3 following USP26 knockout in osteoblasts (Figure 6I,J).

Histone H3 lysine 18 lactylation (H3K18LA) has been reported to positively regulate the transcription of its target genes [55]. Therefore, we sought to determine whether decreased H3K18LA promotes the expression of FSTL1 by inhibiting the expression of its repressor in Usp26^−/−^ osteoblasts. The core of the FSTL1 regulatory network is the FSTL1 transcript. The stability of the FSTL1 transcript is regulated by RNA‐binding proteins such as KH‐type splicing regulatory protein (KSRP). In specific disease conditions, such as squamous cell carcinoma, reduced expression of KSRP can lower the alternative splicing of FSTL1 mRNA, resulting in increased transcription of FSTL1 mRNA over microRNA 198 [56]. Therefore, we sought to determine whether the decrease in H3K18LA levels inhibited the transcriptional expression of KSRP in Usp26^−/−^ osteoblasts. To test this, we analyzed the mRNA and protein expression of KSRP in WT and Usp26^−/−^ osteoblasts. The results revealed significant downregulation of KSRP expression at both the mRNA and protein levels after USP26 knockout (Figure 6K,L). Furthermore, immunofluorescence staining demonstrated decreased KSRP expression in osteoblasts of Usp26 cKO mice (Figure 6M,N). Additionally, the reduction in H3K18LA, decrease in KSRP expression, and increase in FSTL1 expression in Usp26^−/−^ osteoblasts could be reversed by exogenous lactate stimulation (Figure 6O–R).

Next, we sought to determine whether H3K18LA promotes KSRP transcription by enriching its promoter regions. With the H3K18LA ChIP‐seq of GSE156675, we found that H3K18LA was enriched in the promoter region of KSRP mRNA (promoter: Chr19:6424812‐6426311) (Figure 6S). Furthermore, ChIP‐qPCR experiments revealed that H3K18LA was significantly enriched in KSRP promoter regions. This suggests that H3K18LA facilitates KSRP mRNA transcription by binding to its promoter. (Figure 6T). Taken together, these findings indicated that a reduction in H3K18LA levels in the promoter region of Fstl1 hinders the transcriptional expression of KSRP in Usp26^−/−^ osteoblasts. This, in turn, enables the translation of FSTL1 by decreasing the KSRP‐mediated alternative splicing of FSTL1 mRNA.

### 2.6 Activating the Osteoblastic USP26 Pathway Through EV‐Based Bone‐Targeting Drug Effectively Prevents Insulin Resistance‐Induced Multi‐Organ Fibrosis

After determining that the loss of USP26 in osteoblasts leads to impaired bone formation, IR, and multi‐organ fibrosis, we investigated whether activating USP26 in osteoblasts could promote bone formation and alleviate fibrosis induced by IR. Engineered EVs with targeted modifications have a significant therapeutic potential for the treatment of various disorders due to their biocompatibility and stability. E‐selectin, which is expressed in bone vascular niches, plays a critical role in bone formation [57, 58]. EVs modified with Golgi glycoprotein 1 (GLG1), a ligand of E‐selectin, serve as an effective bone‐targeted delivery platform and show promise for the treatment of bone diseases [59]. The NIH/3T3 cell line overexpressing Glg1 and Usp26 was used. To track the proteins, mCherry and EGFP tags were incorporated into GLG1 and USP26, respectively. EVs were harvested from 3T3 cell supernatants by ultracentrifugation and subsequently used for both in vitro and in vivo applications.

**FIGURE 7:**
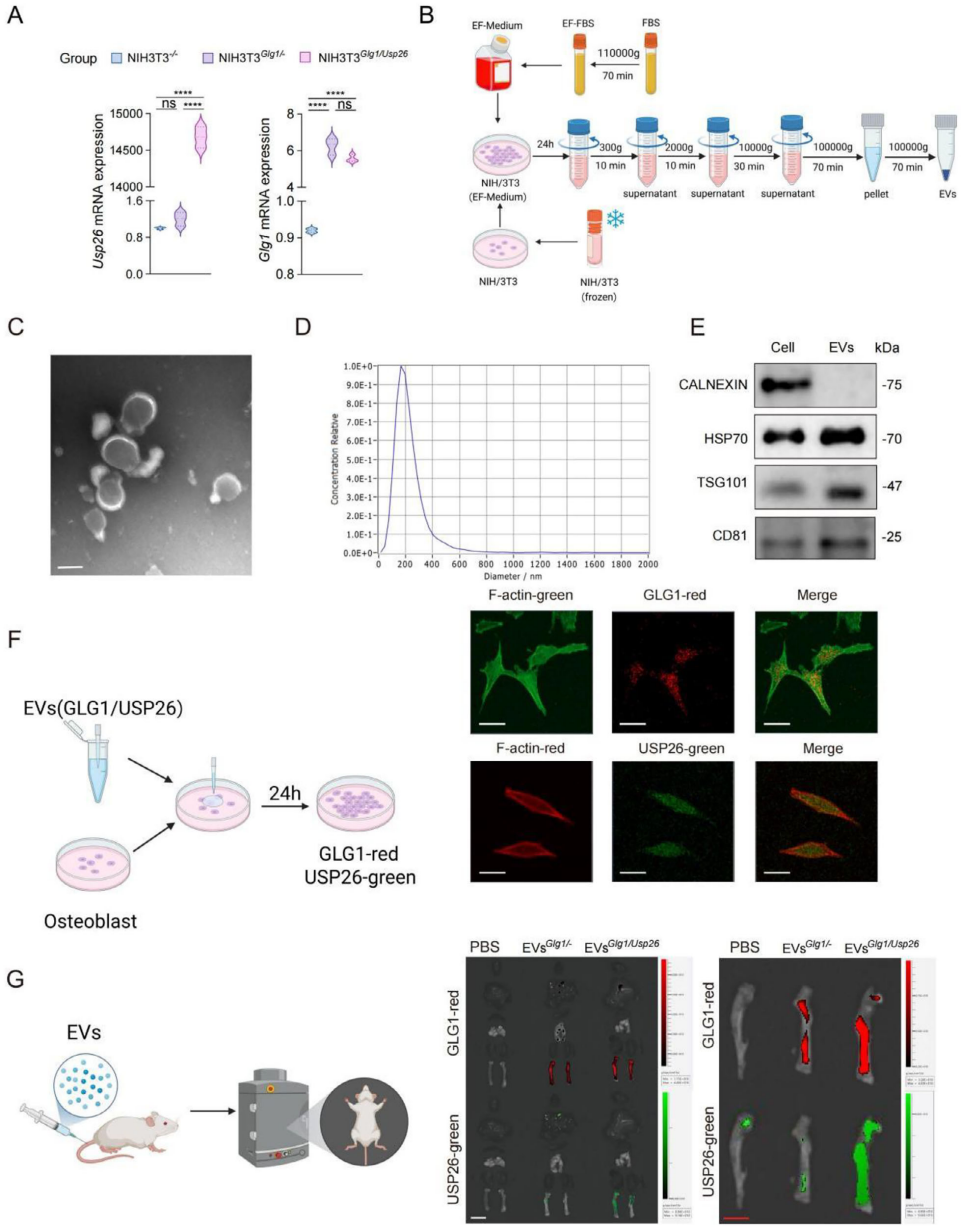
Construction of engineered bone‐targeting EVs with overexpression of Usp26 and/or Glg1. (A) qPCR analysis of Usp26 and Glg1 mRNA levels in 3T3 cells. *n *= 3 in each group. (B) Schematic diagram of EVs isolation via ultracentrifugation. (C) Electron microscopy images of EVs. *n *= 5. Scale bars: 100 nm. (D) Size distribution profile of EVs. *n *= 5 (E) Western blot analysis of CALNEXIN, HSP70, TSG101 and CD81 expression in 293T cells and EVs. *n* = 3 in each group. (F) Cellular uptake of EVs. *n *= 5 in each group. Scale bars: 20 µm. (G) Targeting specificity analysis of EVs. *n *= 6 in each group. White scale bars: 10 mm; red scale bars: 4 mm. Data are presented as mean ± SEM. Statistical significance was analyzed using one‐way ANOVA. ****p < 0.0001.

RNA analyses indicated the successful establishment of our overexpression system. Both GLG1 and USP26 were highly expressed in the stable cell lines overexpressing GLG1 and/or USP26 (Figure 7A). We then used differential centrifugation to isolate EVs (Figure 7B). The in vitro identification and characterization results showed that EVs had a typical round or elliptical cup‐shaped structure, with particle sizes mainly distributed in the range of 0 to 200 nm (Figure 7C,D). The EVs expressed the markers TSG101, CD81, and HSP70 (Figure 7E). Additionally, real‐time PCR results showed that EVs successfully carried Glg1 and Usp26 (Figure S8). Co‐culture with osteoblasts showed that EVs carrying Glg1 and Usp26 could enter osteoblasts to promote the transcriptional expression of USP26 in these cells (Figure 7F). To further determine whether GLG1/USP26‐EVs can target the bone in vivo, mice were intravenously injected with GLG1/USP26‐EVs and subjected to biophotonic imaging analysis. The results showed a significant aggregation of EVs in the femur tissue, indicating that our GLG1/USP26‐EVs could target the skeletal system (Figure 7G). Collectively, these data demonstrate that GLG1/USP26‐EVs can target the bone and upregulate the expression of USP26 in osteoblasts.

**FIGURE 8:**
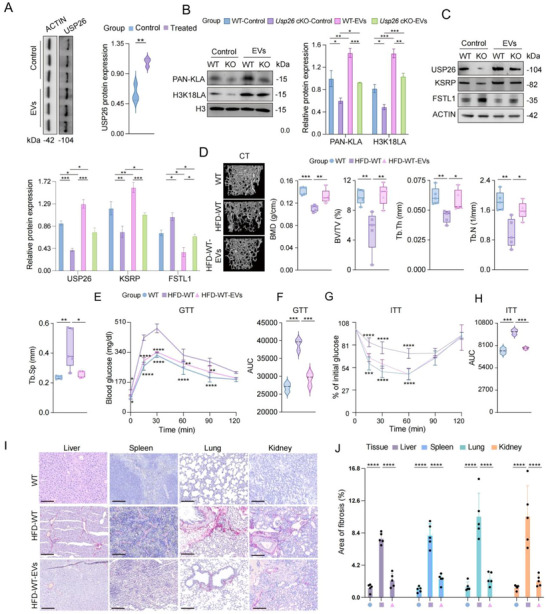
Engineered EVs alleviate HFD‐induced IR and fibrosis in mice. (A) Western blot analysis and quantification of USP26 protein expression in osteoblasts treated with PBS or engineered EVs. *n *= 3 in each group. (B) Western blot analysis of PAN‐KLA and H3K18LA protein expression in osteoblasts from Usp26 cKO mice and their littermate controls treated with PBS or engineered EVs. *n *= 3 in each group. (C) Western blot analysis of USP26, KSRP, and FSTL1 protein expression in osteoblasts from Usp26 cKO mice and their littermate controls treated with PBS or engineered EVs. *n *= 3 in each group. (D) Representative micro‐CT images of femurs from WT, HFD‐induced mice, and HFD‐induced mice with EVs injection via the tail vein. Quantitative analysis of trabecular bone parameters: BV/TV, Tb. N, Tb. Th, Tb. Sp, and BMD. *n *= 5 in each group. (E, F) Glucose tolerance test (GTT) and area under the curve (AUC) in WT, HFD‐induced mice, and HFD‐induced mice with EVs injection. *n *= 5 in each group. (G, H) Insulin tolerance test (ITT) and AUC in WT, HFD‐induced mice, and HFD‐induced mice with EVs injection. *n *= 5 in each group. (I) Sirius Red staining of liver, spleen, lung, and kidney tissues from WT, HFD‐induced mice, and HFD‐induced mice with EVs injection. *n *= 5 in each group. Scale bars: 100µm. (J) Quantitative analysis of Sirius Red staining. *n *= 5 in each group. Data are presented as mean ± SEM. Statistical significance was analyzed using two‐tailed Student's *t*‐test in (A), one‐way ANOVA in (B), (C), (D), (F), (H), and (J), and two‐way ANOVA in (E) and (G). *p < 0.05, **p < 0.01, ***p < 0.001, ****p < 0.0001.

We then aimed to determine whether GLG1/USP26‐EVs could upregulate the downstream pathways of USP26 in osteoblasts. Osteoblasts were treated with USP26/GLG1‐EVs for 24h, and western blotting results demonstrated that engineered EVs enhanced the expression of USP26 (Figure 8A). Additionally, GLG1/USP26‐EVs promoted total histone lactylation, H3K18LA, and KSRP expression, ultimately inhibiting the secretion of FSTL1 in osteoblasts (Figure 8B,C).

To assess the therapeutic effects of GLG1/USP26‐EVs in vivo, we used high‐fat diet (HFD)‐induced IR and multi‐organ fibrosis models. Our data revealed that 21 weeks of HFD significantly decreased USP26 expression in osteoblasts, which in turn promoted bone loss, glucose intolerance, IR, and fibrosis in organs such as the liver, spleen, lungs, and kidneys. However, the intravenous injection of GLG1/USP26‐EVs significantly blocked these processes (Figure 8D–J; Figure S9A,B). Overall, these findings demonstrate that activation of the osteoblastic USP26 pathway through EV‐based bone‐targeting drugs effectively prevents IR‐induced multi‐organ fibrosis in an HFD model.

### 2.7 Activating the Osteoblastic USP26 Pathway Through Compression Loading Prevents Insulin Resistance‐Induced Multi‐Organ Fibrosis

Next, we aimed to determine whether activating the osteoblastic USP26 pathway through a noninvasive method could effectively prevent IR‐induced multi‐organ fibrosis. Running, a common form of physical therapy, is widely used in the treatment of chronic diseases because of its safety and convenience [19]. Physical activity can also provide compressive loading to osteoblasts, promoting bone formation and metabolic functions. In this study, we found that compression loading using the Flexcell Compression System increased both the mRNA and protein levels of USP26 in osteoblasts. Therefore, we further investigated whether activation of the osteoblastic USP26 pathway through compression loading could prevent IR‐induced multi‐organ fibrosis.

**FIGURE 9:**
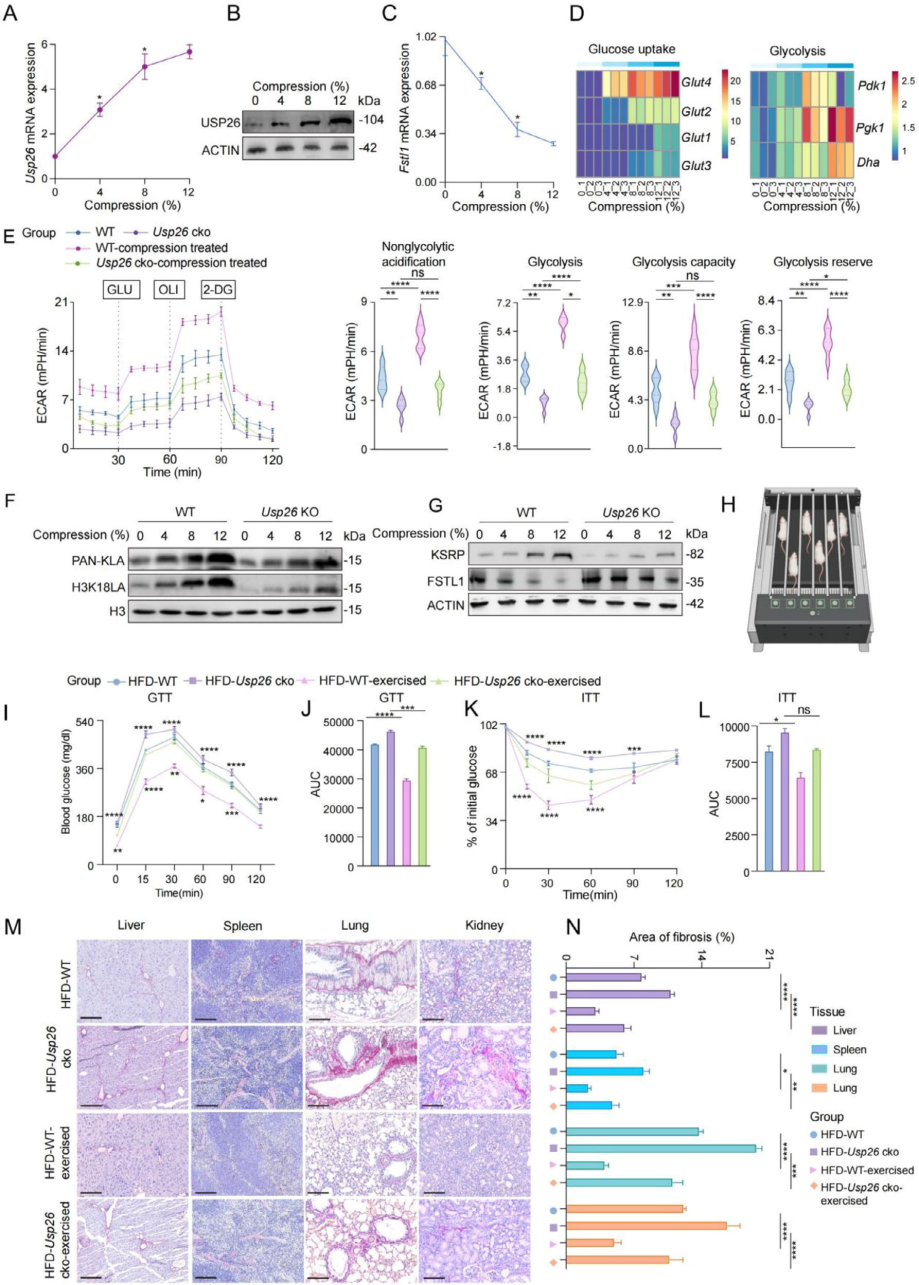
Activating the osteoblastic USP26 pathway through compression loading prevents IR‐induced multiorgan fibrosis. (A) qPCR analysis of USP26 mRNA in osteoblasts under different compression. *n *= 3 in each group. (B) Western blot analysis of USP26 protein expression in osteoblasts under different compression conditions. *n *= 3 in each group. (C) qPCR analysis of the Fstl1 gene mRNA under different compression. *n *= 3 in each group. (D) qPCR analysis of glucose uptake and glycolysis‐related genes in osteoblasts under different compression. *n *= 3 in each group. (E) ECAR analysis of osteoblasts from Usp26 cKO mice and their Littermate controls under compression. *n *= 5 in each group. (F) Western blot analysis of PAN‐KLA and H3K18LA protein expression in osteoblasts from Usp26 cKO mice and their Littermate controls under different compression. *n *= 3 in each group. (G) Western blot analysis of KSRP and FSTL1 protein expression in osteoblasts from Usp26 cKO mice and their Littermate controls under different compression. *n *= 3 in each group. (H) Schematic diagram of mice exercise (I, J) GTT and corresponding area under the curve (AUC) for both Usp26 cKO mice and their littermate controls under high‐fat diet (HFD) conditions, with or without daily running exercise. *n *= 5 in each group. (K, L) ITT and corresponding area under the curve (AUC) for both Usp26 cKO mice and their littermate controls under high‐fat diet (HFD) conditions, with or without daily running exercise. *n *= 5 in each group. (M) Sirius red staining of liver, spleen, lung, and kidney from both Usp26 cKO mice and their littermate controls under high‐fat diet (HFD) conditions, with or without daily running exercise. Scale bars: 100 µm. *n *= 5 in each group. (N) Quantitative analysis of Sirius red. *n *= 5 in each group. Data are presented as mean ± SEM. Statistical significance was analyzed using one‐way ANOVA in (A), (C), (D), (E), (J), (L), and (N), two‐way ANOVA in (I) and (K). *p < 0.05, **p < 0.01, ***p < 0.001, ****p < 0.0001.

In response to compression loading, upregulation of USP26 expression (Figure 9A,B), significant downregulation of FSTL1 expression (Figure 9C), and a significant increase in the expression of glucose uptake‐ and glycolysis‐related genes (Figure 9D) were observed in osteoblasts. Furthermore, ECAR assay results indicated that compression loading enhanced glycolysis in osteoblasts, dependent on USP26 expression (Figure 9E). In line with the findings on glucose uptake and glycolysis, USP26 knockout resulted in significant inhibition of compression loading‐induced total histone lactylation and H3K18LA (Figure 9F). In addition, impaired promotion of KSRP and inhibition of FSTL1 expression by compression loading upon deletion of Usp26 were also detected (Figure 9G). These findings suggest that compression loading induces USP26 expression in osteoblasts and potentially inhibits FSTL1 expression by activating the H3K18LA‐KSRP pathway.

To investigate whether the activation of the osteoblastic USP26 pathway through compression loading could prevent IR‐induced multi‐organ fibrosis in vivo, an HFD‐induced IR and multi‐organ fibrosis model was utilized. This was followed by an 8‐week running exercise regimen to apply compression loading to the osteoblasts. Our findings revealed that running increased USP26 expression in the osteoblasts of HFD mice (Figure S10A,B) and significantly improved bone mass (Figure S10C,D). Additionally, it significantly enhanced glucose clearance from the bloodstream, as shown by the GTT results (Figure 9I,J), and increased insulin sensitivity, as indicated by ITT results (Figure 9K,L). Furthermore, Sirius red staining demonstrated that HFD‐induced multi‐organ fibrosis was alleviated after running. However, this effect was partially reversed by USP26 knockout in osteoblasts (Figure 9M,N). In conclusion, these results collectively indicate that activation of the osteoblastic USP26 pathway through compression loading can prevent IR‐induced multi‐organ fibrosis in the HFD model.

## 3 Discussion

**FIGURE 10:**
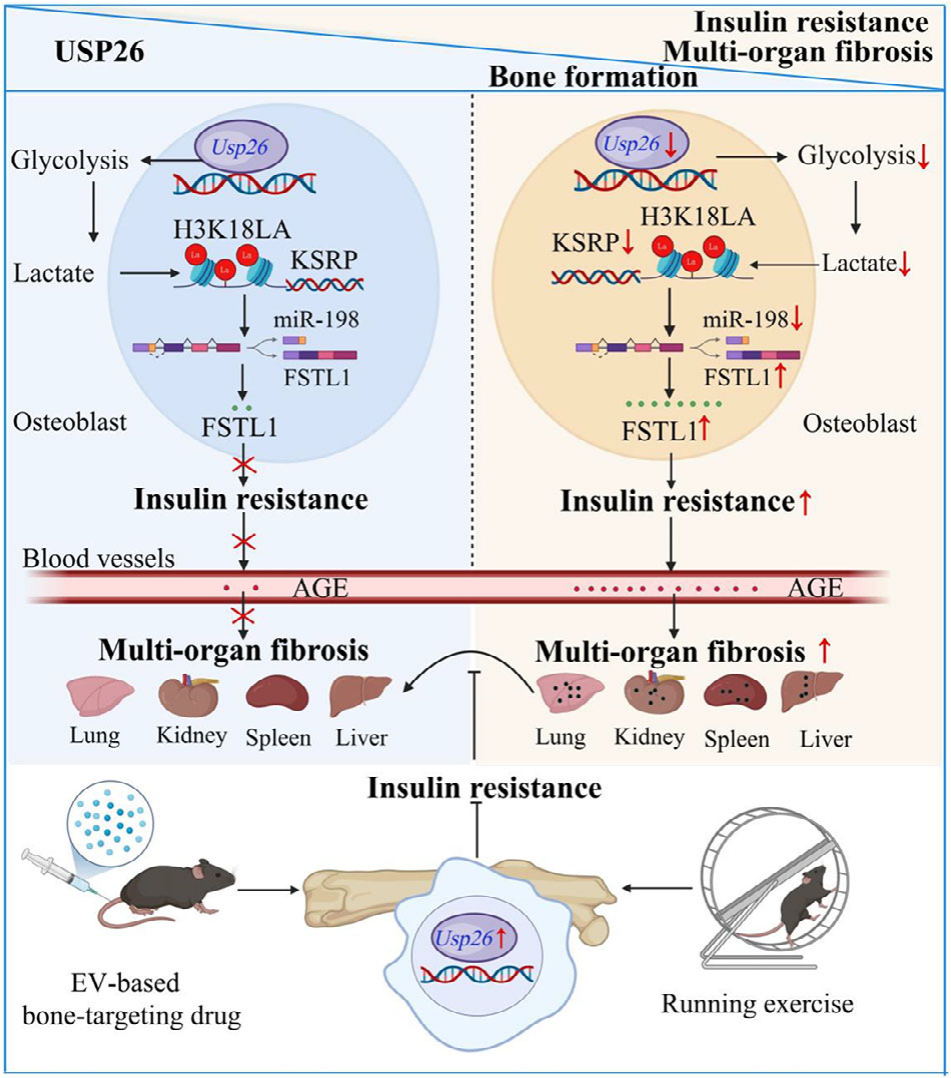
Schematic of activating the osteoblastic USP26 pathway alleviates multiorgan fibrosis by decreasing IR. The loss of USP26 in osteoblasts results in decreased bone formation, as well as multi‐organ fibrosis associated with IR. Mechanistically, the absence of USP26 reduces glycolysis and lactate accumulation, leading to decreased H3K18LA in the promoter region of KSRP. This results in lower transcription of KSRP and diminished alternative splicing of FSTL1 mRNA by KSRP. Consequently, the elevated FSTL1 contributes to IR and elevated blood glucose levels. This leads to the buildup of AGEs in the blood, ultimately leading to multi‐organ fibrosis. Furthermore, activating the USP26 pathway specifically in osteoblasts through EV‐based bone‐targeting drug or compression loading can effectively prevent multi‐organ fibrosis induced by IR.

Multi‐organ fibrosis is commonly regarded as a metabolic disorder [6, 60]. This study revealed that the loss of USP26 in osteoblasts leads to decreased bone formation and multi‐organ fibrosis associated with IR. Reduced H3K18LA/KSRP increases FSTL1 expression by reducing the alternative splicing of FSTL1 mRNA, which in turn causes IR and the accumulation of AGEs in the blood, resulting in multi‐organ fibrosis. Furthermore, activating the USP26 pathway, specifically in osteoblasts, through EV‐based bone‐targeting drugs or mechanical loading can effectively prevent multi‐organ fibrosis induced by insulin IR (Figure 10). Thus, this study revealed that osteoblast dysfunction is a key inducer of metabolism‐related fibrosis and underscores osteoblastic USP26 as a promising therapeutic target for addressing multi‐organ fibrosis associated with IR.

As an endocrine organ, bone cells exhibit active metabolic activities and have autocrine and paracrine effects [61, 62]. Among the most active cells in the skeletal system, osteoblasts secrete various bone‐derived factors that regulate organ functions [63], maintain normal metabolic activity, and participate in various pathological processes [64, 65]. For instance, osteoblasts secrete slit guidance ligand 2 to promote the browning of white adipose tissue [66], while OCN alleviates nonalcoholic fatty liver disease in mice through GPRC6A [67]. Fibroblast growth factor 23, another factor secreted by osteoblasts, regulates phosphorus metabolism [68]. Recent studies have shown that interleukin 11 from osteoblasts and osteocytes regulates fat metabolism [69]. In addition, osteoblast‐derived lipocalin 2 helps maintain glucose homeostasis by inducing insulin secretion and improving glucose tolerance and insulin sensitivity [70]. Moreover, we have previously reported that activation of the osteoblastic HIF‐1α pathway partially alleviates the symptoms of streptozotocin (STZ)‐induced type 1 diabetes mellitus via RegIIIγ [30]. In this study, we demonstrated that the loss of USP26 in osteoblasts results in decreased bone formation and multi‐organ fibrosis associated with IR. This discovery deepens our understanding of the endocrine functions of bone and underscores the potential significance of the osteoblast dysfunction–metabolism disorder‐multi‐organ fibrosis axis as a crucial pathological pathway.

Bone tissue degeneration is a typical degenerative disease in the elderly [71], and its pathological mechanism is mainly characterized by a significant decrease in osteoblast activity with age, leading to a slowing of the bone formation rate and continuous loss of bone mass [72]. It is worth noting that the aging process not only affects the skeletal system but also triggers a series of metabolic function disorders, such as IR [73] and abnormal glucose metabolism [74], thereby increasing the risk of developing metabolic diseases such as type 2 diabetes [75]. Recent studies have suggested that skeletal degeneration may be a risk factor for the development of diabetes mellitus, as the treatment of osteoporosis with denosumab can significantly reduce diabetes [76]. Considering the significant role of metabolic disorders in fibrosis progression, the susceptibility to metabolism‐related fibrosis may increase with decreased osteoblast function and skeletal degeneration. Interestingly, our previous reports have shown that with aging and a decline in the osteogenic differentiation function of osteoprogenitor cells, the expression of USP26 gradually decreases [32]. In the present study, we further revealed that the loss of USP26 in osteoblasts inhibited osteoblast osteogenic differentiation and led to a decrease in bone mass, accompanied by IR and multi‐organ fibrosis. Moreover, activating the USP26 pathway, specifically in osteoblasts, through EV‐based bone‐targeting drugs or mechanical loading can effectively promote bone formation and prevent multi‐organ fibrosis induced by IR. Therefore, these findings suggest that reduced USP26 expression in osteoblasts could serve as a predictor of bone loss and metabolism‐related fibrosis, and also indicate that USP26 may be a new therapeutic target for treating metabolic fibrotic diseases by promoting bone health.

Investigation of the molecular mechanisms underlying multi‐organ fibrosis caused by USP26 deficiency revealed that, in addition to obesity and IR, USP26 cKO also led to elevated expression and release of FSTL1 in osteoblasts. This finding is consistent with increased levels of FSTL1 detected in the blood of patients with obesity and IR. FSTL1 has been shown in numerous studies to regulate fibrosis by affecting the function of macrophages [77], influencing cell glycolysis, and regulating blood glucose levels [78, 79]. Our findings suggest that the deletion of USP26 in osteoblasts induces fibrosis in multiple organs without significantly altering the number of myeloid and T cells in the bloodstream. Therefore, the marked upregulation of FSTL1 expression resulting from USP26 deletion in osteoblasts is more likely to induce fibrosis in multiple organs through IR than through the immune microenvironment.

The skeletal muscle is the primary organ responsible for glucose uptake in response to insulin stimulation [80]. During insulin‐resistant states, skeletal muscles are among the first tissues to be affected. Our results showed that skeletal muscle tissue from USP26 cKO mice exhibited fibrosis, as demonstrated by Masson's and Sirius Red staining (Figure S11A,B). However, the expression levels of inflammatory markers such as IL‐1β, IL‐6, and TNF‐α were not elevated (Figure S11C). Additionally, we observed decreased protein levels of tyrosine–phosphorylated IRS‐1 (Tyr612) and AKT (Ser473), both positive regulators of the insulin signaling pathway, in the skeletal muscle tissues of USP26 cKO mice (Figure S11D,E). These findings indicate that USP26 knockout in osteoblasts can induce IR and cause fibrosis in skeletal muscle.

Although organ‐specific pathways play important roles in fibrosis, certain similarities exist. A key common feature of fibrosis is the activation and proliferation of myofibroblasts, which produce and deposit excessive amounts of ECM, directly leading to scar formation [81, 82]. AGEs and their receptor (RAGE) are important factors that induce myofibroblast differentiation and trigger fibrotic changes [83, 84]. For example, studies have shown that treating hepatic stellate cells with AGEs significantly upregulates the expression of α‐SMA, which can be reversed by treatment with soluble RAGE (sRAGE) [85]. Similarly, in studies related to renal fibrosis, treatment of renal tubular cells with AGEs also increases the expression of α‐SMA and collagen type I (COL1) [86]. To further investigate the role of AGEs in the development of fibrosis in the liver, spleen, lungs, and kidneys, we treated hepatic stellate cells (Figure S12A), spleen fibroblastic reticular cells (Figure S12B), pulmonary fibroblasts (Figure S12C), and renal fibroblasts (Figure S12D) with phosphate‐buffered saline, AGEs, or AGEs plus sRAGE for 24 h. We found that α‐SMA and COL1 protein levels were significantly elevated in the AGE‐treated groups, but this increase could be reversed by sRAGE. These findings, together with the observation that administration of AG to Usp26 cKO mice led to a significant reduction in multi‐organ fibrosis (Figure 3G), strongly demonstrated the activating effect of AGEs on hepatic stellate cells, pulmonary fibroblasts, splenic fibroblastic reticular cells, and renal fibroblasts.

AGEs can promote the progression of fibrosis by increasing the non‐enzymatic crosslinking of collagen. Collagen crosslinking occurs through two main mechanisms: enzymatic crosslinking, which is regulated by lysyl hydroxylase and lysyl oxidase, and non‐enzymatic crosslinking, which is induced by AGEs through glycation or oxidation. AGEs participate in non‐enzymatic glycation reactions with the amino groups on collagen, forming irreversible covalent cross‐linkages that make the collagen fibers stiffer and more resistant to degradation, thereby promoting tissue fibrosis and aging. The dysregulation of collagen crosslinking is a common feature of fibrosis progression [87]. Increased AGE‐mediated non‐enzymatic collagen crosslinking has been confirmed in multiple studies and models [88, 89], and may represent a common mechanism by which AGEs contribute to multi‐organ fibrosis.

Dynamic changes in glucose levels analyzed by PET‐CT revealed that IR prevented glucose uptake by peripheral organs, such as the heart, liver, spleen, lungs, and kidneys. These organs are most frequently affected by IR and metabolic syndrome [90, 91], and the development of fibrosis in these tissues is closely associated with hyperglycemia and AGE accumulation [92, 93, 94]. Our results show that osteoblastic USP26 deletion significantly induced fibrosis in the liver, spleen, lungs, and kidneys. However, we did not observe typical extensive fibrosis in the heart as observed in other organs. This finding may be explained as follows. Although IR and AGE accumulation are known to contribute to diabetic cardiomyopathy and myocardial fibrosis, USP26 knockout in osteoblasts results in increased expression and peripheral circulation of FSTL1. Previous studies have demonstrated that FSTL1 reverses myocardial fibrosis and exerts protective effects on cardiac structures by reducing collagen deposition. For example, FSTL1 can alleviate doxorubicin‐induced cardiotoxicity by upregulating Nrf2 to inhibit apoptosis and oxidative stress [95], whereas its loss of function leads to the activation of the SMAD3 signaling pathway and the promotion of venous wall and atrial fibrosis [96]. Moreover, the FSTL1–USP10–Notch1 signaling axis has been shown to protect against cardiac dysfunction in diabetic mice by inhibiting myocardial fibrosis [97]. Additionally, the application of epicardial patches containing human FSTL1 effectively improved cardiac function and suppressed myocardial fibrosis in porcine and murine models of myocardial infarction [98, 99]. Therefore, the protective action of FSTL1 may offset the effects of AGEs on the differentiation of cardiac fibroblasts into myofibroblasts, preventing significant fibrosis in the heart.

We fully recognize that FSTL1, as a known pro‐fibrotic factor, exerts its function in close association with the activation of the transforming growth factor‐beta (TGF‐β) signaling pathway. In the early stages of our study, we conducted an in‐depth exploration of the potential contribution of the FSTL1–TGF‐β axis to multi‐organ fibrosis in Usp26 cKO mice. However, this classical pathway alone cannot fully account for all the phenotypes observed in our study, particularly some of the apparent contradictions. Extensive studies have demonstrated that TGF‐β plays a crucial anabolic role in skeletal homeostasis. For example, Piezo1‐mediated mechanotransduction in M2 macrophages has been shown to significantly enhance bone formation by promoting the secretion and activation of TGF‐β1 [100]. Another study indicated that TGF‐β1 promotes the osteogenic differentiation of mesenchymal stem cells by activating a series of biochemical and biophysical signals, such as regulating integrin‐mediated focal adhesion formation and mechanosensing, and acts in concert with pathways such as bone morphogenetic protein [101]. These compelling lines of evidence suggest that upregulation of TGF‐β is generally associated with increased bone formation. This is in clear conflict with the pronounced reduction in bone mass observed in Usp26 cKO mice.

Given that the strong association between osteoporosis and diabetes is widely recognized, we infer that, in the specific pathological context revealed by our study, the metabolic disturbances characterized by systemic IR and hyperglycemia driven by USP26 deficiency may outweigh any potential anabolic effect of local TGF‐β signaling on bone formation. Therefore, while the TGF‐β pathway may still contribute to target organ fibrosis, the USP26–FSTL1–IR–AGEs axis that we propose offers a more comprehensive and causally coherent explanatory framework for the coexistence of osteoporosis, systemic IR, and metabolic multi‐organ fibrosis in our mouse model.

While studying the molecular mechanism underlying the increased expression of FSTL1 owing to the loss of USP26, we found that the absence of USP26 diminished glycolysis and lactate accumulation in osteoblasts, resulting in a decrease in H3K18LA. This decrease in H3K18LA in the promoter region of KSRP reduces KSRP transcription, leading to reduced alternative splicing of FSTL1 mRNA, ultimately resulting in elevated FSTL1 expression. Although the link between USP26 knockout and reduced histone lactylation was indirect, a dramatic decrease in the expression of glucose transporter 1 (Glut1), glucose transporter 2 (Glut2) and glucose transporter 3 (Glut3) was observed in USP26 KO osteoblasts. Analysis of RNA‐seq data (Figure S13A) from osteoblasts of both WT and USP26 cKO mice, together with biochemical experiments (Figure S13B), revealed that reduced expression of kruppel‐like factor 15 (Klf15) may contribute to the decreased levels of Glut1, Glut2, and Glut3 in USP26 KO osteoblasts, as restoration of Klf15 expression significantly rescued the reduced expression of Gluts caused by USP26 deficiency. Additionally, previous studies have indicated that AMPK phosphorylation can indirectly promote GLUT expression [102], and our experimental data demonstrated a significant reduction in AMPK phosphorylation following USP26 knockdown. Therefore, we speculated that decreased AMPK activity may also contribute to the reduced expression of GLUTs in USP26 cKO mice.

A decrease in glycolysis inhibits osteoblast differentiation and bone formation, which may also explain why impaired osteoblastic activity and bone formation were observed in osteoblasts with Usp26 deletion. Additionally, a decrease in glycolysis can decrease glucose utilization in osteolineage cells [103], potentially directly inhibiting systemic glucose clearance, promoting glucose tolerance, and resulting in sustained increases in blood glucose levels and peripheral fat accumulation [104]. These effects may also contribute to the obesity, high blood sugar levels, and decreased glucose clearance observed in USP26 cKO mice.

In summary, our study demonstrates that activation of the osteoblastic USP26 pathway alleviates multi‐organ fibrosis by decreasing IR and suggests that osteoblastic USP26 is a promising therapeutic target for addressing multi‐organ fibrosis associated with metabolic disorders.

## 4 Method and Materials

### 4.1 Mice

USP26^flox/flxo^ mice (T001875) and Fstl^flox/flxo^ mice (T018356) were purchased from GemPharmatech in the C57BL/6J mouse strain. USP26^flox/flox^ mice were crossed with Ocn‐cre mice to generate osteoblast conditional Usp26 knockout mice (USP26^flox/flox^‐Ocn‐Cre^+/−^, Usp26 cKO). Fstl^flox/flxo^ mice were crossed with Usp26 cKO mice to generate osteoblast conditional Usp26 and Fstl1 knockout mice (USP26^flox/flox^/Fstl^flox/flxo^‐Ocn‐Cre^+/−^, Usp26/Fstl1 dKO). Genotyping of all mice was performed using the One‐Step Mouse Genotyping Kit (Vazyme, #PD101, China) from the mouse tail. All mice were housed in a pathogen‐free animal facility at LENSCI Biotechnology (Kunshan) Co., LTD, under a 12‐h light and 12‐h dark cycle, with free access to food and water.

### 4.2 Human Bone Tissues and Serum Acquisition

Bone tissue acquisition: Following patient‐informed consent, non‐lesioned bone samples were obtained during joint replacement surgery, subjected to cryogenic grinding in liquid nitrogen, and directly cryopreserved for downstream analyses. All specimens underwent standardized quality control measures.

Serum collection: After 8 h of fasting, 6–8 mL of whole blood was drawn from the antecubital vein into BD SST II serum tubes (367958), allowed to clot at room temperature for 30 min, and centrifuged at 3000 g for 10 min at 4°C. The supernatant serum was aliquoted and stored at −80°C for subsequent experiments.

### 4.3 Ethical Statement

This study strictly adhered to and directly followed the guidelines for animal care issued by the Ministry of Science and Technology of the People's Republic of China **[IACUC protocol number: 20250311‐0311‐01]**. The procedures experimental adhered procedures to adhered 3Rs principles (Replacement, Reduction, Refinement) through careful design to minimize the animal number usage such and as anesthesia were to employed reduce to animal suffering minimize animal suffering, and humane euthanasia to the greatest extent possible. This study is dedicated to honoring the intrinsic value of animal life, with their contributions directed exclusively toward irreplaceable scientific research purposes.

For human serum and bone tissues acquisition, written informed consent was obtained, and ethics approval was granted by the Ruijin Hospital Ethics Committee, Shanghai Jiao Tong University School of Medicine, with the project number of 2017‐2‐20.

### 4.4 Cell Culture

About the isolation and culture of osteoblasts, primary osteoblasts were isolated from 3‐day‐old USP26 cKO and littermate control neonatal mice. After excising the epidermis and muscle tissue from the skulls, the specimens were digested in a 37°C water bath with 80 mg/mL Type I collagenase (Gibco, 17100017) for 10 min, followed by removal of the digestion solution. This step was repeated once. After discarding the digested material, the skulls were minced and subjected to an additional digestion with 80 mg/mL Type I collagenase for 20 min. The resulting digested mixture was centrifuged at 1500 rpm for 5 min to pellet the cells. This digestion process was repeated three times. The cells harvested from all three digestions were pooled together and plated into 60 × 15 mm culture dishes (Corning, 430166). The cells were cultured in α‐MEM (Gibco, 12571048) supplemented with 100 IU/mL penicillin, 100 mg/mL streptomycin (Gibco, 15140122), and 10% fetal bovine serum (Gibco, 10099141). Upon reaching the second passage, the isolated osteoblasts were used for subsequent experiments.

For the culture of 3T3 cells, the thawed cells were seeded onto 150 mm culture dishes (Servicebio, CCD‐150) and cultured in high‐glucose DMEM medium (Servicebio, G4512‐500mL) supplemented with 100 IU/mL penicillin, 100 mg/mL streptomycin (Gibco, 15140122), and 10% fetal bovine serum (FBS) (Gibco, 10099141), for subsequent EVs isolation.

### 4.5 Metabolic Studies and Bioassays

For the GTT, mice were fasted overnight, and blood glucose was measured via tail clipping and recorded as the baseline level. A glucose solution (2g/kg body weight) was then administered via intraperitoneal injection. Blood glucose levels were monitored at 5, 15, 30, 60, and 120 min post‐injection using a Roche Accu‐Chek Excellence Gold Collection glucometer.

For the insulin tolera ITT, mice were fasted for 6 h, and baseline blood glucose was similarly measured via tail clipping. Insulin (0.75 U/kg body weight; CAS 207748‐29‐6) was injected intraperitoneally, and blood glucose levels were assessed at 15, 30, 60, 90, and 120 min post‐injection.

### 4.6 Neutralization of AGE in Mouse Blood

Aminoguanidine (MCE, HY‐B1041A) was used to neutralize AGE in mouse blood. The specific method was as follows: Aminoguanidine was dissolved in saline. Then, the aminoguanidine solution was administered to 22‐week‐old Usp26 cKO mice via oral gavage (100 mg/kg), once daily for two weeks. After two weeks, the mice were anesthetized with isoflurane, euthanized, and blood samples were collected for serum preparation to perform subsequent experiments.

### 4.7 HFD‐Induced Mouse Model of Insulin Resistance and Fibrosis

Four‐week‐old Usp26 cKO mice and their littermate control mice were fed a purified HFD with 60% fat‐derived calories (XTHF60, Synergistic Bio) for 4 weeks. Subsequently, they received daily intraperitoneal injections of a 55 mg/kg streptozotocin (STZ; R014987) solution prepared in citrate buffer (R050384) for 5 consecutive days. Following the injections, the mice continued to receive their corresponding diets and were maintained under these conditions for a total of 16 weeks.

### 4.8 EVs Isolation

Fetal bovine serum (Gibco, 10099141) was centrifuged at 110 000× g for 70 min to obtain EV‐depleted serum. A complete culture medium was prepared by supplementing DMEM (LD1111‐500) with 100 IU/mL penicillin, 100 mg/mL streptomycin (Gibco, 15140122), and 10% EVs‐depleted serum. NIH‐3T3 cells overexpressing USP26 and Glg1 were cultured in this medium until reaching 75% confluency, followed by an additional 24‐h incubation. The supernatant was collected and sequentially centrifuged at 300×g for 10 min to remove cellular debris, then at 2000×g for 10 min (twice), followed by 10 000×g for 30 min. Finally, the supernatant was ultracentrifuged at 100 000×g for 70 min. The resulting pellet was resuspended and subjected to a second ultracentrifugation at 100 000×g for 70 min to purify EVs overexpressing USP26 and Glg1.

### 4.9 Experimental Validation of Cellular Internalization and Bone‐Targeting Capacity of EVs

For the cellular internalization, EVs were co‐incubated with osteoblasts plated on glass‐bottom dishes (density: 2 × 10⁴ cells/cm^2^) for 24 h (37°C, 5% CO_2_). After removing uninternalized EVs, cells were washed three times with PBS, fixed with 4% paraformaldehyde, stained with phalloidin (Servicebio, IF488/IF555) for cytoskeletal visualization and DAPI for nuclear labeling, and finally imaged using a confocal microscope (ZEISS, Germany) to detect intracellular fluorescent signals.

For the bone‐targeting capacity of EVs, EVs overexpressing USP26 and GLG1 were administered via tail vein injection to 24‐week‐old WT mice. Four hours post‐injection, mice were anesthetized with isoflurane, followed by the collection of the heart, liver, lungs, kidneys, femurs, and tibiae. Tissues were immediately placed into the Tanon ABL‐X6 imaging system, and signal intensities across organs were quantified using dedicated analysis software.

### 4.10 Application of Bone‐Targeting EVs

Model mice established with a HFD were administered bone‐targeting EVs overexpressing USP26 and GLG1 via tail vein injection. Injections were performed every three days for a total duration of 4 weeks. The mice were closely monitored throughout the administration period. Following the completion of the 4‐week treatment regimen, blood glucose levels were measured, and metabolic test GTT and the ITT, were conducted. Finally, mice were anesthetized with isoflurane and euthanized. Tissues, including the femur, serum, liver, spleen, lung, and kidney, were collected for subsequent experiments.

### 4.11 Western Blot

Lyse cell samples in RIPA buffer on ice for 20 min. Collect the lysed cells and centrifuge at 14 000 g to remove debris. Measure protein concentration using a BCA Protein Assay Kit (P0009, Beyotime). Pre‐identify and exclude samples with low protein yield. Separate samples containing 10 µg total protein by SDS‐PAGE on 10% gels and transfer to PVDF membranes (EMD Millipore Corporation, USA). After blocking with 5% skim milk in Tris‐buffered saline containing 1‰ Tween (TBST), incubate membranes overnight at 4°C with antibodies against OSTERIX (28694‐1‐AP,Proteintech), RUNX2 (ab23981, Abcam), USP26 (ab230226, Abcam), ALP (ab307726, Abcam), β‐actin (#58169, CST), AMPK (A12718, ABclonal), P‐AMPK (AP‐0432, ABclonal), FSTL1 (20182‐1‐AP, proteintech), PAN‐KLA (PTM‐1401RM, PTM BIO), H3K18LA (PTM‐1406RM, PTM BIO), H3K9LA (PTM‐1419RM, PTM BIO), H3K14LA (PTM‐1414RM, PTM BIO), H4K8LA (PTM‐1415RM), H4K12LA (PTM‐1411RM), H3 (PTM‐1002RM, PTM BIO), KSHRP (#13398, CST), calnexin (ab22595, Abcam), HSP70 (ab2787, Abcam), CD81 (ab79559, Abcam), TSG101 (ab125011, Abcam), Phospho‐AKT (Ser473) (66444‐1‐Ig, Proteintech), AKT (10176‐2‐AP, Proteintech),Phospho‐IRS1 (Tyr612) (ZRB09432, Merck), IRS1(17509‐1‐AP, Proteintech), JNK (51153‐1‐AP, Proteintech), Phospho‐JNK (Thr183/Tyr185) (60666‐1‐lg,Proteintech), Phospho‐IRS1 (Ser307) (85238‐1‐RR, Proteintech), Alpha smooth muscle actin (14395‐1‐AP, Proteintech) and Collagen Type I (14695‐1‐AP, Proteintech,). Wash membranes three times with TBST, then incubate with horseradish peroxidase‐conjugated secondary antibodies (Jackson). Visualize antibody–antigen complexes using Immobilon reagent (Millipore).

### 4.12 Real‐Time Quantitative RT‐PCR

Total RNA was isolated using TRIzol reagent (Invitrogen, USA). Reverse transcription of 1µg total RNA into cDNA was performed using the PrimeScript Reverse Transcription Kit (#RR036A, Takara, Japan). Gene expression analysis was conducted using the SYBR Premix Ex Taq Kit (Takara, Japan) on an ABI 7500 Real‐time PCR system (Applied Biosystems, USA), with β‐actin as the endogenous reference gene for normalization. Target gene‐specific primer sequences are detailed in Table S1. Low‐concentration RNA samples were excluded through pre‐experimental quality control screening (RNA integrity number ≥7.0). All procedures followed standardized protocols outlined in the manufacturer's instructions.

### 4.13 Micro‐Computed Tomography (Micro‐CT)

Micro‐CT analysis of murine left femurs was performed as previously described. Following fixation in 4% paraformaldehyde, specimens were scanned using a Skyscan 1172 micro‐CT scanner (Aartselaar, Belgium) in accordance with guidelines from the American Society for Bone and Mineral Research (ASBMR). The system parameters included a 10 µm isotropic voxel size, 50 keV voltage, 500 µA current, and 0.7° rotation step. Region‐of‐interest (ROI) definitions were established for trabecular analyses. The trabecular ROI spanned 1 mm proximal to the distal growth plate and extended 1 mm toward the diaphysis. 2D gray‐scale representations of trabecular structures were reconstructed from relative cross‐sections. Evaluated trabecular parameters included bone mineral density (BMD, g/cm^3^), bone volume fraction (BV/TV, %), trabecular thickness (Tb.Th, mm), trabecular separation (Tb.Sp, mm), and trabecular number (Tb.N, 1/mm)

### 4.14 ELISA

Strictly following the manufacturer's instructions, we measured serum levels of FSTL1 (AF43265‐A, AiFang Biological), osteocalcin (AF9285‐A, AiFang Biological), insulin (AF2579‐A, AiFang Biological), TG (A3010S0476D, BioTNT), and AGE (#EM0716, FineTest) in mice, while FSTL1 (#EH0142, FineTest) and AGE (#EH0622, FineTest) were assayed in human subjects; samples with insufficient protein yield were pre‐screened and excluded prior to analysis.

### 4.15 Extracellular Acidification Rate (ECAR) Assay

Cells were counted and plated at a density of 10 000–30 000 cells per well. Diluted cell suspensions were gently dispensed into XF96 cell culture microplates (180 µL per well). XF96 sensor cartridges were hydrated overnight in calibration solution, and drugs were prepared according to the manufacturer's protocol. After overnight incubation, cell morphology and confluency were verified via microscopic examination. Seahorse XF assay medium was pre‐warmed in a 37°C water bath. Cell culture medium was aspirated, and cells were washed twice with 180 µL of assay medium per well. Finally, 175 µL of assay medium was added to each well, and cell detachment status was confirmed. The microplate was incubated in a non‐CO_2_ incubator for 60 min. Drugs (glucose, oligomycin, and 2‐DG) were sequentially loaded into designated injection ports of the sensor cartridge. The culture plate and sensor cartridge were then loaded into the Seahorse XFe96 Analyzer (Seahorse Bioscience, USA) for ECAR measurement.

### 4.16 Radioactive Glucose Tracer

Biodistribution assessment of 18F‐FDG and micro‐PET imaging was performed in 24‐week‐old mice (*n *= 5). After overnight fasting, mice were anesthetized with isoflurane and intravenously injected with 18F‐FDG (dose: µCi = body weight [g] × 16). All PET images were acquired using a micro‐PET/CT scanner (PINGSENG, China) at 15 min, 30 min, 45 min, 60 min, and 70 min post‐injection. PET data were reconstructed using the Inveon Research Workplace (PINGSENG, China) with a 3D ordered subset expectation maximization/maximum a posteriori (OSEM3D/MAP) algorithm without scatter correction. Regions of interest (ROI) were delineated on PET images, and the percentage of injected dose per gram (%ID/g) in corresponding organs was calculated.

### 4.17 Histomorphometry

Decalcified bone tissues were embedded in paraffin, while soft tissues such as liver, spleen, lung, kidney, and skin were fixed in 4% paraformaldehyde and embedded in paraffin. Sections of 5 µm thickness from both bone and soft tissues were stained with H&E using standard protocols. All histomorphometric parameters were calculated and reported according to the recommendations proposed by the nomenclature committee of the ASBMR. For undecalcified bone specimens, femoral bones were dehydrated and embedded in methyl methacrylate. Sections of 5 µm thickness were prepared using a Leica RM2235 microtome and stained with von Kossa.

For Masson staining, soft tissues (liver, spleen, lung, and kidney) were fixed in 4% paraformaldehyde. After thorough fixation and dewaxing, Masson staining (BA4079B, Baso) was performed strictly following the manufacturer's instructions. For Sirius red staining, soft tissues (liver, spleen, lung, kidney) were adequately fixed in 4% paraformaldehyde, and staining was conducted on 6–7 µm‐thick sections using the corresponding kit (BA4356, Baso) as per the protocol. Fibrosis‐related quantification and analysis for Masson and Sirius red staining were performed using ImageJ, with one section analyzed per sample and a total of five samples assessed.

### 4.18 Immunostaining

After dewaxing, sections were treated with 3% H_2_O_2_ (room temperature, 15 min) to quench endogenous peroxidase activity, followed by blocking of nonspecific binding sites with 5% bovine serum albumin (BSA, room temperature, 10 min). Primary antibodies were applied and incubated overnight at 4°C, including USP26 (ab230226, abcam) and α‐SMA (GB111364, Servicebio). The second day, sections were incubated with biotinylated secondary antibodies at room temperature, and signals were amplified and visualized using the streptavidin–biotin–peroxidase complex (ABC method). Nuclei were counterstained with hematoxylin, and images were captured using an optical microscope (Zeiss AXIO system). Negative controls were included throughout the experiment to exclude nonspecific staining caused by technical factors. Statistical analysis was performed using ImageJ software.

For immunofluorescence, sections were blocked with 5% goat serum (containing 0.3% bovine serum albumin and 0.1% sodium azide) for 1 h in a humidified chamber to prevent drying after dewaxing. Then sections were rinsed three times with PBS (5 min each). Primary antibodies, including FSTL1 (20182‐1‐AP, Proteintech), OCN(ab93876, Abcam), and KSRP (ab150393, Abcam), were diluted and applied to sections, followed by overnight incubation at 4°C in a humidified chamber. The second day, sections were washed three times with PBS (5 min each) to remove unbound primary antibodies. Fluorescently labeled secondary antibodies were then added and incubated in the dark at room temperature for 1 h in a humidified chamber. For nuclear counterstaining, sections were stained with 4′,6‐diamidino‐2‐phenylindole (DAPI, 1 µg/mL) in the dark for 5 min, followed by three PBS washes (5 min each). Finally, sections were mounted with anti‐fade mounting medium and observed/imaged using a fluorescence or confocal microscope. The intensity and distribution of fluorescent signals were quantitatively analyzed using ImageJ software.

### 4.19 RNA Sequencing

Following total RNA extraction from osteoblasts and construction of mRNA sequencing libraries, transcriptome data were subjected to FPKM quantification using StringTie and Ballgown. Significantly differentially expressed genes (DEGs) were identified via the edgeR package, with subsequent systematic pathway analysis employing KEGG enrichment, Gene Ontology (GO) annotation, and Gene Set Enrichment Analysis (GSEA) to delineate dysregulated pathways between control and Usp26 conditional knockout (cKO) mouse osteoblasts.

### 4.20 Proteomics

Fresh femoral tissues from Usp26 cKO mice and their littermate controls were decalcified in 0.5M EDTA, pulverized in liquid nitrogen, and total proteins extracted using high‐intensity lysis buffer (4% SDS/100 mm Tris‐HCl). Protein concentrations were quantified by BCA assay. Proteins were reduced with dithiothreitol (DTT), alkylated with iodoacetamide (IAA), and digested overnight with Trypsin/Lys‐C, followed by desalting and purification of peptides. The peptides were separated and purified by high‐performance liquid chromatography (HPLC). Purified peptides were subjected to mass spectrometry analysis, with subsequent database searching for protein identification. Bioinformatics tools were employed to analyze differentially expressed proteins in femoral bone tissue between Usp26 cKO and WT littermate control mice.

### 4.21 ChIP

The ChIP protocol was performed using a commercial assay kit (Millipore Sigma, Burlington, MA). Briefly, cells were fixed with formaldehyde, and the reaction was quenched with 125 mm glycine. Cell lysis was carried out using a buffer containing 5 mm PIPES (pH 8), 85 mm KCl, 0.5% NP‐40, 20 mm sodium butyrate, and protease/phosphatase inhibitors (2 mm PMSF, 20 mm NaF, 1X aprotinin, 0.1 mg/mL leupeptin, and 2 mm Na_3_VO_4_). Chromatin was fragmented to 100–500 bp using a Bioruptor sonicator (Diagenode, Denville, NJ). For immunoprecipitation, 200 µg of fragmented chromatin was incubated overnight at 4°C with either 3 µg of H3K18LA antibody (PTM BIO, PTM‐1406RM) or negative control anti‐IgG (CST, #5415) to form immune complexes, followed by coupling to Protein A beads. After treatment with proteinase K and RNase A, crosslinks were reversed (65°C, 12–16 h).

For ChIP‐qPCR, the precipitated DNA was purified using the QIAquick PCR Purification Kit (Qiagen, Valencia, CA). Both input samples and purified H3K18LA‐ChIP‐DNA were analyzed by qPCR, with immunoprecipitated sample values normalized against input DNA. Primers targeting putative H3K18LA‐binding regions (6425171‐6425191) on the KSRP promoter were designed using DNASTAR Lasergene 15.2 Core Suite (Madison, WI) and Primer‐BLAST, with sequences provided in Table S2. Immunoprecipitated DNA was quantified by RT‐qPCR, and fold changes were calculated using the ΔΔCt method with normalization to % input, presented as fold enrichment relative to control mouse IgG.

For ChIP‐seq, Libraries were constructed using the NEBNext Ultra II DNA Library Prep Kit, followed by quantification with Qubit and quality assessment on Agilent Bioanalyzer. Sequencing was performed on the Illumina NovaSeq 6000 platform with 150 bp paired‐end reads. Raw sequencing data underwent quality control via FastQC, aligned to the reference genome using Bowtie2, processed for peak calling with MACS2, and subjected to motif enrichment analysis through HOMER.

### 4.22 Mouse Daily Running Exercise

Following successful establishment on a HFD, mice underwent acclimation training prior to an 8‐week treadmill exercise protocol: Weeks 1–2 at 10 m/min speed, 0° incline for 20 min/day (5 days/week); Weeks 3–6 at 12 m/min, 5° incline for 30 min/day (5 days/week); Weeks 7–8 at 15 m/min, 5° incline for 30 min/day (5 days/week). Then, mice underwent GTT and ITT. Subsequently, animals were anesthetized with isoflurane and euthanized for the collection of liver, spleen, lung, kidney, and bone tissues for downstream analyses. Throughout the training period, body weights were monitored weekly with exercise intensity adjustments based on weight dynamics. Exercise sessions were continuously observed via video surveillance to ensure stable performance and exclude exhausted subjects.

### 4.23 Isolation of Mouse Splenic Fibroblastic Reticular Cells

Spleen tissues were thoroughly minced and digested in a solution containing 0.2 mg/mL Collagenase P (Roche) and 0.1 mg/mL DNase I (Roche) at 37°C for 15 min. The resulting single‐cell suspension was collected and washed three times with PBS. Red blood cells were subsequently removed using red blood cell lysis buffer. The remaining splenocytes were cultured in α‐MEM medium supplemented with 10% fetal bovine serum and 1% penicillin/streptomycin to allow adherent growth. After 48 h, non‐adherent cells were removed by washing, and the adherent cells were expanded to passage 2 for subsequent experiments. Finally, splenic fibroblastic reticular cells (FRCs, phenotype: CD45^−^CD31^−^gp38⁺) were sorted by flow cytometry, typically achieving >95% purity.

### 4.24 Fibroblast Treatment

Mouse hepatic stellate cells (CP‐M041, Procell System, China), mouse pulmonary fibroblasts (CP‐M006, Procell System, China), mouse renal fibroblasts (CP‐M069, Procell System, China), and splenic fibroblastic reticular cells were treated with PBS, AGEs, or AGEs combined with sRAGE (RD272590100, Amyjet Scientific, China) for 24 h. Total cellular protein was then extracted for subsequent Western blot analysis.

### 4.25 Administration of FSTL1‐Neutralizing Antibody

Usp26 cKO mice received intraperitoneal injections of either an FSTL1‐neutralizing antibody (NM050408, Abmart, China) or a control IgG1 antibody (50µg per mouse per injection). The injections were given at 2‐day intervals for a total of three injections. Tissues, including liver, spleen, lungs, and kidneys, were collected 4 days after the final injection for Masson's trichrome staining to assess fibrosis.

### 4.26 Statistical Analysis

All experimental data are expressed as mean ± standard error of the mean (SEM). Statistical evaluation of intergroup differences was performed using two‐tailed *t*‐tests; comparisons among multiple groups were analyzed by one‐ or two‐way analysis of variance (ANOVA) with Bonferroni correction for multiple comparisons using GraphPad Prism 9.5 software. Statistical significance was defined as *p* < 0.05.

## Author Contributions

C.L. and L.D conceived and designed the experiments; J.T., W.Y., L.H., L.C., Z.Y., Y.J., and Z.D. performed the experiments; C.L. and J.T. analyzed the data; C.L., G.T., and L.D. contributed reagents/materials/analysis tools; C.L. wrote, reviewed, and revised the manuscript. All authors read and approved the final paper.

## Conflicts of Interest

The authors declare no conflict of interest.

## Supporting information




**Supporting File**: advs73414‐sup‐0001‐SuppMat.docx.

## Data Availability

Research data are not shared.
