# Supplementary material for: Activating the Osteoblastic USP26 Pathway Alleviates Multi‐Organ Fibrosis by Decreasing Insulin Resistance
Source: Adv Sci (Weinh). 2025 Dec 19;13(10):e12424. doi: 10.1002/advs.202512424 (PMC12915124; doi:10.1002/advs.202512424)
Supplement: Supplementary file 1 — Supporting File: advs73414‐sup‐0001‐SuppMat.docx. [file ADVS-13-e12424-s001.docx]

**Supplementary Materials for**

**Activating the Osteoblastic USP26 Pathway Alleviates Multi-Organ Fibrosis by Decreasing Insulin Resistance**

**Authors:**

Jiyuan Tang^1#^, Wenkai Ye^1#^, Liang He^1#^, Zhou Dan^1^, Leilei Chang^1^, Zijie You^1^, Yuanyue Jiang^2,3^, Guoqing Tang^2,3^*, Lianfu Deng^1^*, Changwei Li^1^*

**Affiliations:**

^1^ Department of Orthopedics, Shanghai Key Laboratory for Prevention and Treatment of Bone and Joint Diseases, Shanghai Institute of Traumatology and Orthopedics, Ruijin Hospital, Shanghai Jiao Tong University School of Medicine, 197 Ruijin 2nd Road, Shanghai, 200025, China.

^2^ Department of Orthopedics, Kunshan Hospital of Chinese Medicine, Affiliated Hospital of Yangzhou University, Suzhou, Jiangsu Province, 215300, China.

^3^ Institute of Traumatology and Orthopedics, Kunshan Hospital of Chinese Medicine, Affiliated Hospital of Yangzhou University, Suzhou, Jiangsu Province, 215300, China.

* **Corresponding authors:**

Email: lcw11876@rjh.com.cn (C. Li); 17712485172@yzu.edu.cn (G. Tang); lfdeng@shsmu.edu.cn (L. Deng)

^#^ These authors contributed equally to this work

**This PDF file includes:**

Figures S1 to S13

Table S1 to S2

**
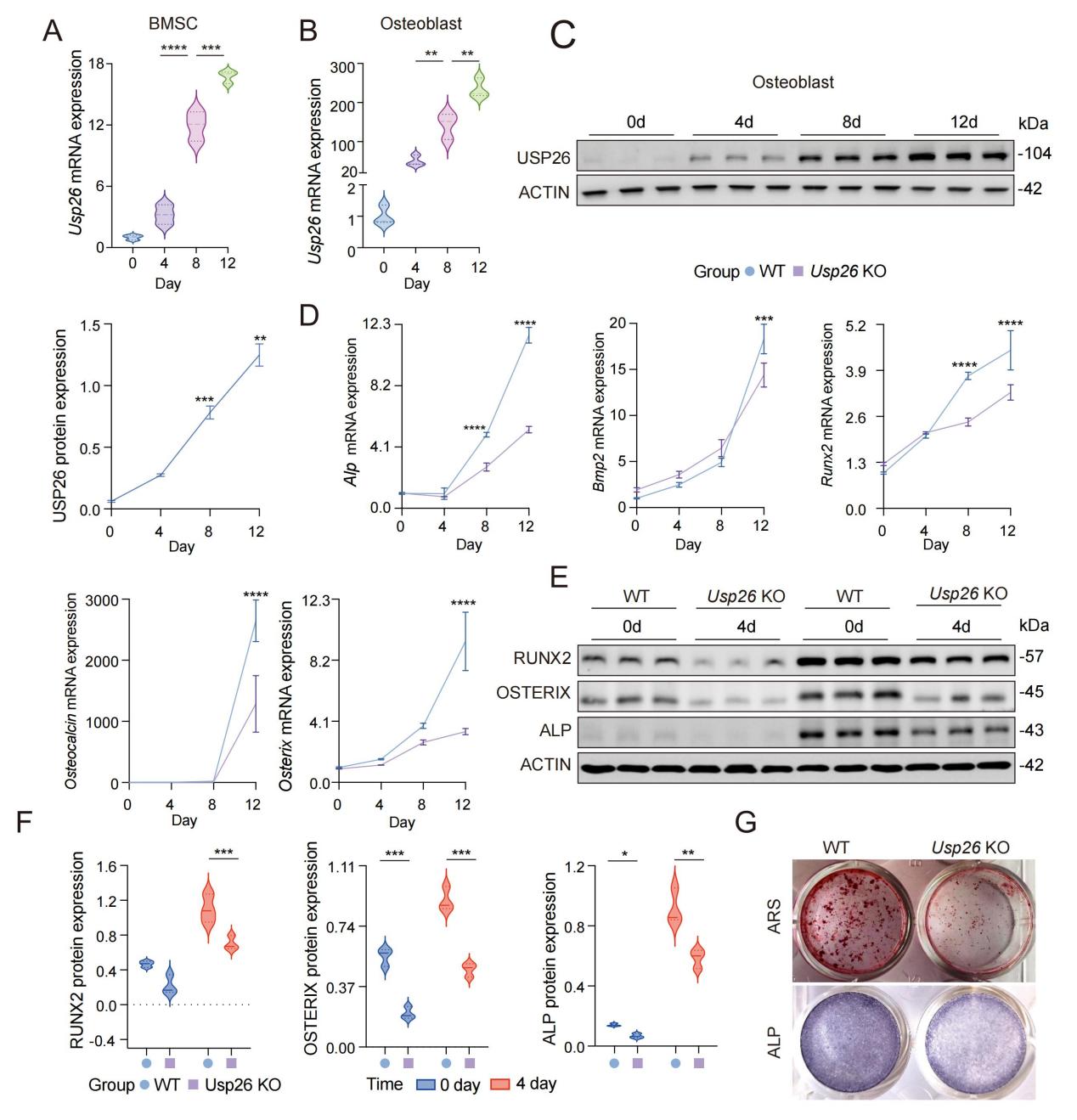
**

**Figure S1. Usp26 knockout impairs the osteogenic capacity of osteoblasts.** (**A**) qPCR analysis of Usp26 expression in BMSCs during osteogenic induction (0, 4, 8, 12 days). n=3 in each group. (**B**) qPCR analysis of Usp26 expression in osteoblasts during osteogenic induction (0, 4, 8, 12 days). n=3 in each group. (**C**) Western blot analysis of USP26 protein expression in osteoblasts during osteogenic induction (0, 4, 8, 12 days). n=3 in each group. (**D**) qPCR analysis of osteogenic markers (Alp, Bmp2, Runx2, Osteocalcin, Osterix) in WT and Usp26 KO osteoblasts during osteogenic induction (0, 4, 8, 12 days). n=3 in each group. (**E**) Western blot analysis of RUNX2, OSTERIX, and ALP protein expression in WT and Usp26 KO osteoblasts during osteogenic induction (0, 4, 8, 12 days). n=3 in each group. (**F**) Statistical analysis of the Western blot results in (E).(**G**) ARS and ALP staining of WT and Usp26 KO osteoblasts. n=3 in each group. Data are presented as mean ± SEM. Statistical significance was determined by one-way ANOVA in (**A**), (**B**) and (**C**). Statistical significance was determined by two-way ANOVA in (**D**) and (**F**). *p < 0.05, **p < 0.01, ***p < 0.001. ****p < 0.0001.

**
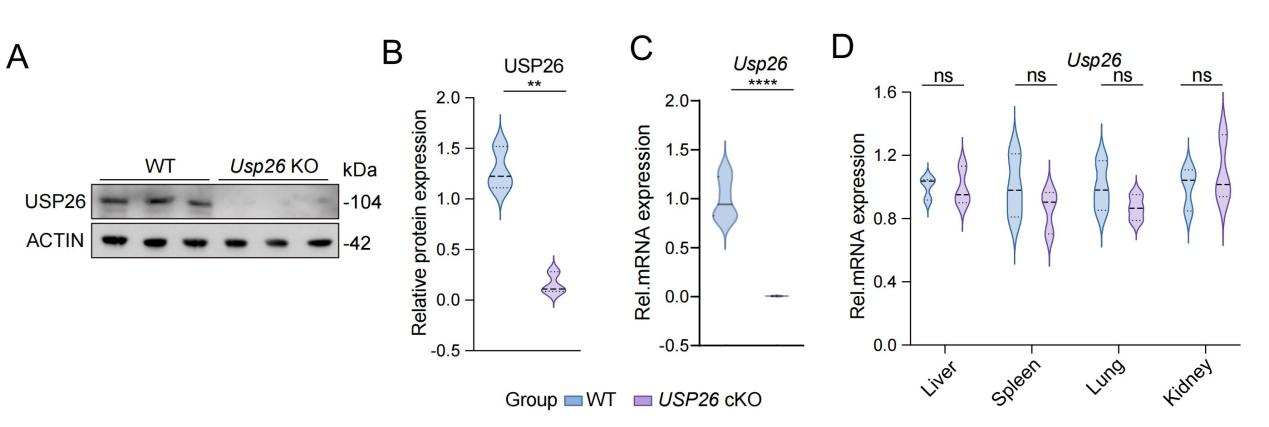
**

**Figure S2. Assessment of USP26 Knockout Efficiency and Specificity.** (**A**) Western blot analysis of USP26 protein levels in osteoblast from Usp26 cKO mice and their littermate controls; n=3 in each group. (**B**) Statistical analysis of Western blot results in (A); n=3 in each group. (**C**) qPCR analysis of Usp26 mRNA levels in osteoblast of Usp26 cKO mice and littermate controls; n=3 in each group. (**D**) qPCR analysis of Usp26 mRNA levels in the liver, spleen, lungs, and kidneys of Usp26 cKO mice and littermate controls; n=3 in each group. Data are presented as mean ± SEM. Statistical significance was determined by two-tailed Student’s t-test. **p < 0.01, ***p < 0.001.


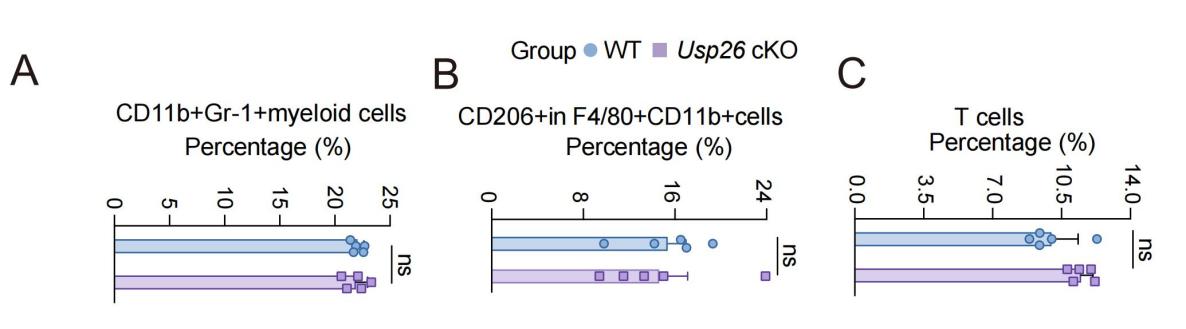


**Figure S3. Usp26 cKO mice do not exhibit significant changes in peripheral blood myeloid cells, macrophages, or T cells.** (**A**) Flow cytometric analysis of CD11b^+^Gr-1^+^ myeloid cells in peripheral blood of Usp26 cKO mice and their littermate controls. n=5 in each group. (**B**) Percentage of CD206^+^ cells in F4/80^+^CD11b^+^ macrophages in peripheral blood of Usp26 cKO mice and their littermate controls. n=5 in each group. (**C**) Percentage of T cells in peripheral blood of Usp26 cKO mice and their littermate controls. n=5 in each group. Data are presented as mean ± SEM. ns represents no significant difference. Statistical significance was determined by two-tailed Student’s t-test.


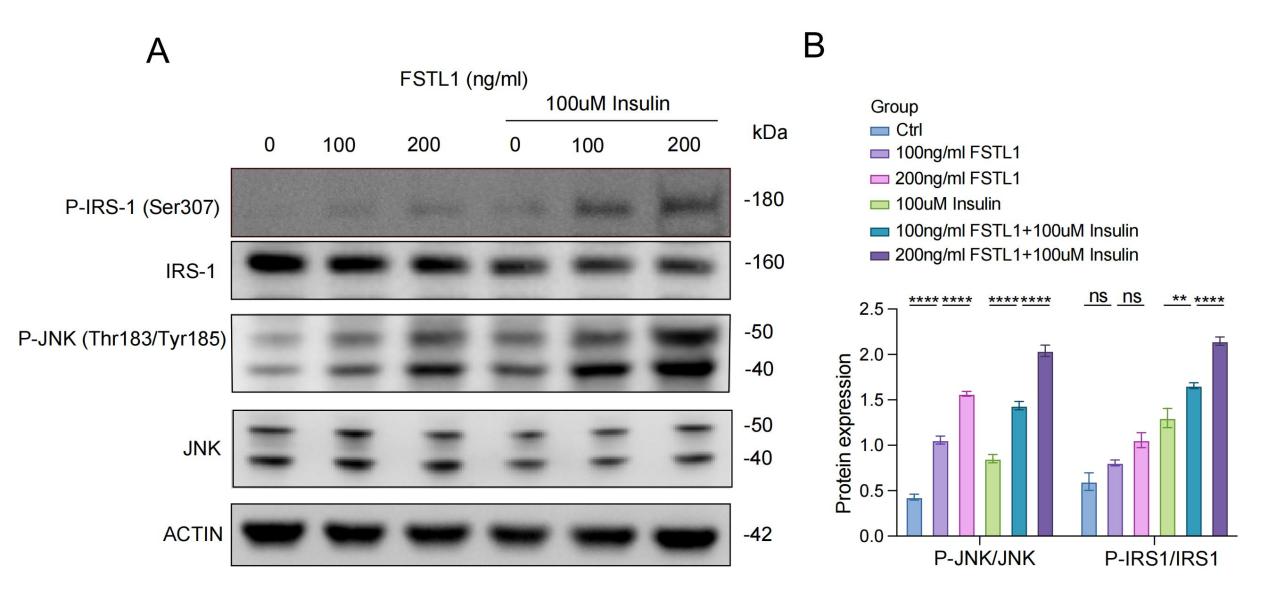


**Figure S4. FSTL1 enhances insulin-induced phosphorylation of JNK and IRS-1 in hepatocytes.** (**A**) Western blot analysis of the phosphorylation of JNK (Thr183/Tyr185) and IRS1 (Ser307) in hepatocytes treated with FSTL1 and/or insulin. (**B**) Statistical analysis of the Western blot results in (A). Data are presented as mean ± SEM. Statistical significance was determined by one-way ANOVA. **p < 0.01, ****p < 0.0001


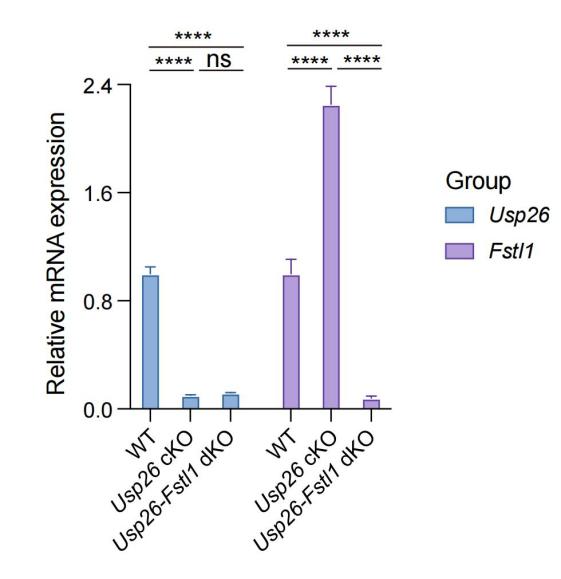


**Figure S5. The knockout efficiency of Usp26 and Fstl1.** qPCR analysis of Usp26 and Fstl1 expression in osteoblasts from WT, Usp26 cKO, and Usp26-Fstl1 dKO mice. n=3 in each group. Data are presented as mean ± SEM. Statistical significance was determined by one-way ANOVA. ****p < 0.0001


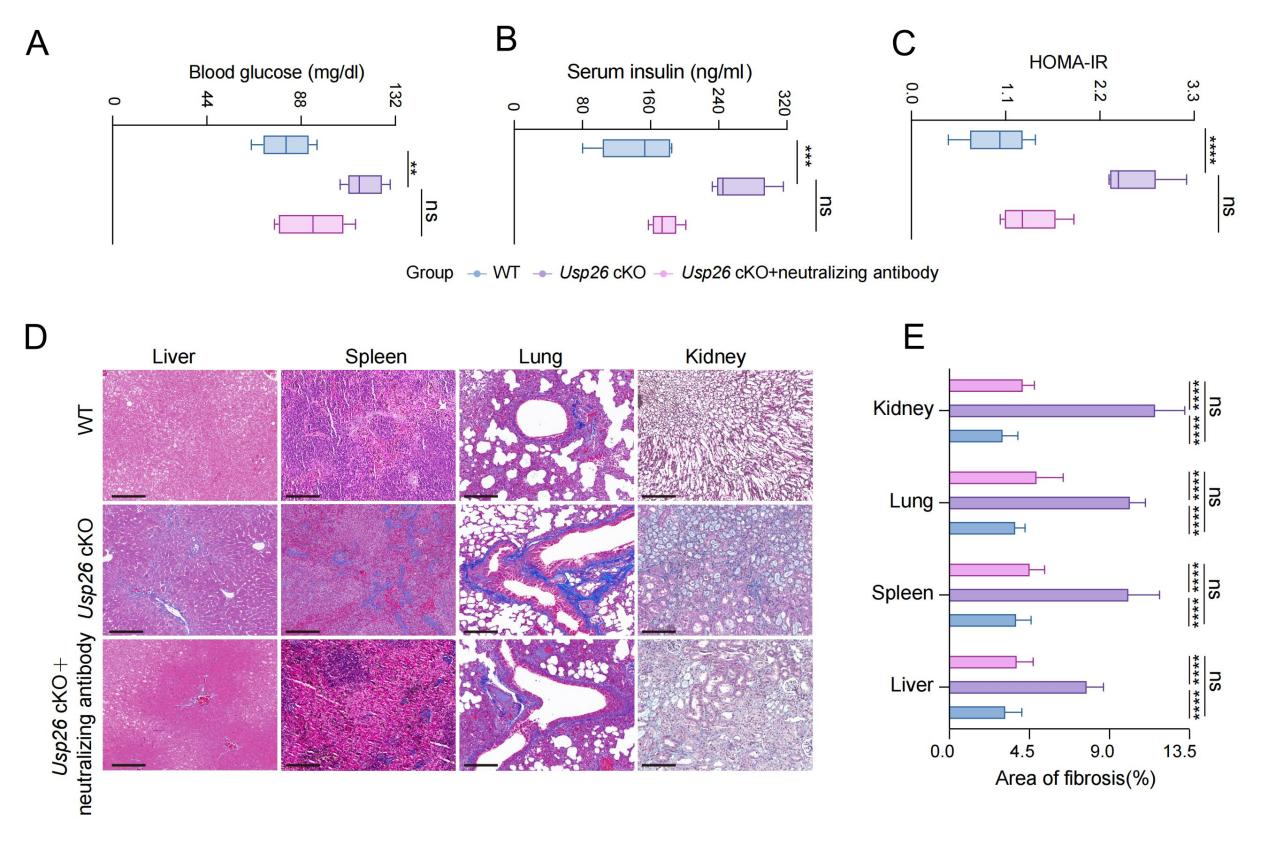


**Figure S6. FSTL1-neutralizing antibody ameliorates insulin resistance and multi-organ fibrosis in Usp26 cKO mice.**(**A**) Fasting blood glucose levels in Usp26 cKO mice, littermate controls, and Usp26 cKO mice treated with FSTL1-neutralizing antibody, n=5 in each group.(**B**) Fasting blood insulin concentration in Usp26 cKO mice, littermate controls, and Usp26 cKO mice administered FSTL1-neutralizing antibody, n=5 in each group.(**C**) HOMA-IR in Usp26 cKO mice, littermate controls, and Usp26 cKO mice treated with FSTL1-neutralizing antibody, n=5 in each group. (**D**) Masson's trichrome staining of liver, spleen, lung, and kidney tissues from Usp26 cKO mice, littermate controls, and Usp26 cKO mice injected with FSTL1-neutralizing antibody, n=5 in each group. Scale bars: 100 μm. (**E**) Quantitative analysis of Masson's trichrome staining in (D), n=5 in each group. Data are presented as mean ± SEM. Statistical significance was determined by two-way ANOVA in (**A**), one-way ANOVA in, (**B**), (**C**), (**D**) and (**F**) **p < 0.01, ***p < 0.001, ****p < 0.0001.


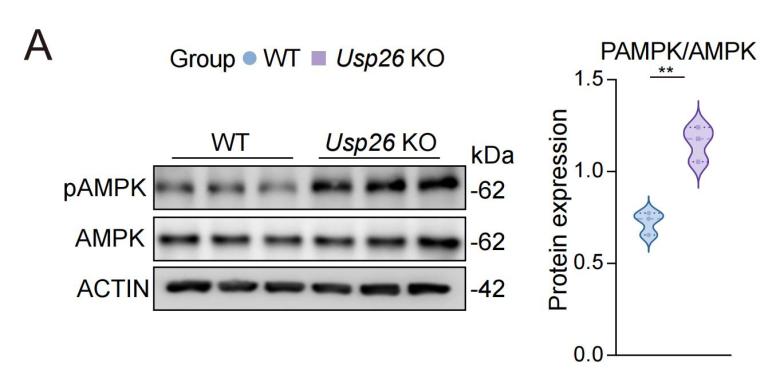


**Figure S7. Western blot analysis the level of p-AMPK in WT and Usp26 KO osteoblasts, with statistical quantification.** Data are presented as mean ± SEM. Statistical significance was determined by two-tailed Student’s t-test. **p < 0.01.


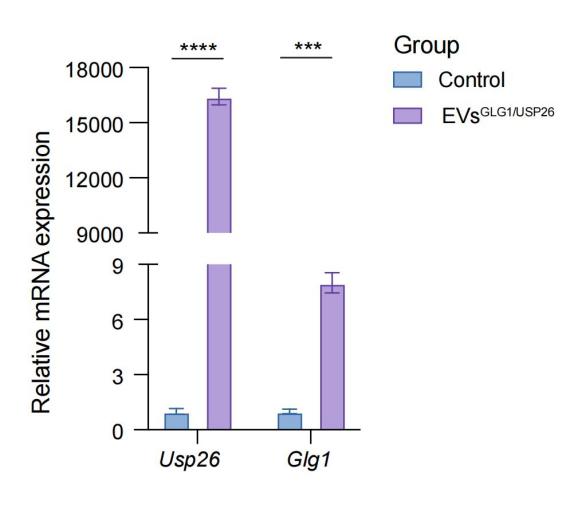


**Figure S8. Engineered EVs successfully loaded with overexpressed USP26 and GLG1.** qPCR analysis of USP26 and GLG1 mRNA levels in GLG1/USP26-EVs. Data are presented as mean ± SEM. Statistical significance was determined by two-tailed Student’s t-test. ***p < 0.001，****p < 0.0001.


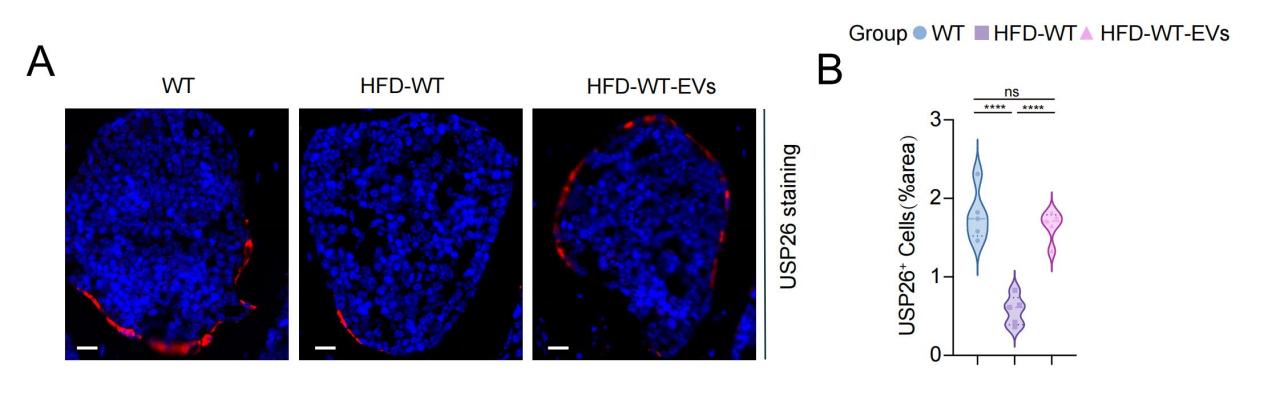


**Figure S9. Engineered exosomes rescued the reduction of USP26 expression in osteoblasts of mice under high-fat diet conditions.** (**A, B**) Immunofluorescence staining of USP26 in femoral trabecular bone from WT mice under: normal diet (WT), high-fat diet (HFD-WT), and HFD followed by intravenous injection of engineered exosomes (HFD-WT-EVs). n=5 in each group. Scale bars: 15μm. Data are presented as mean ± SEM. Statistical significance was determined by one-way ANOVA. ****p < 0.0001.


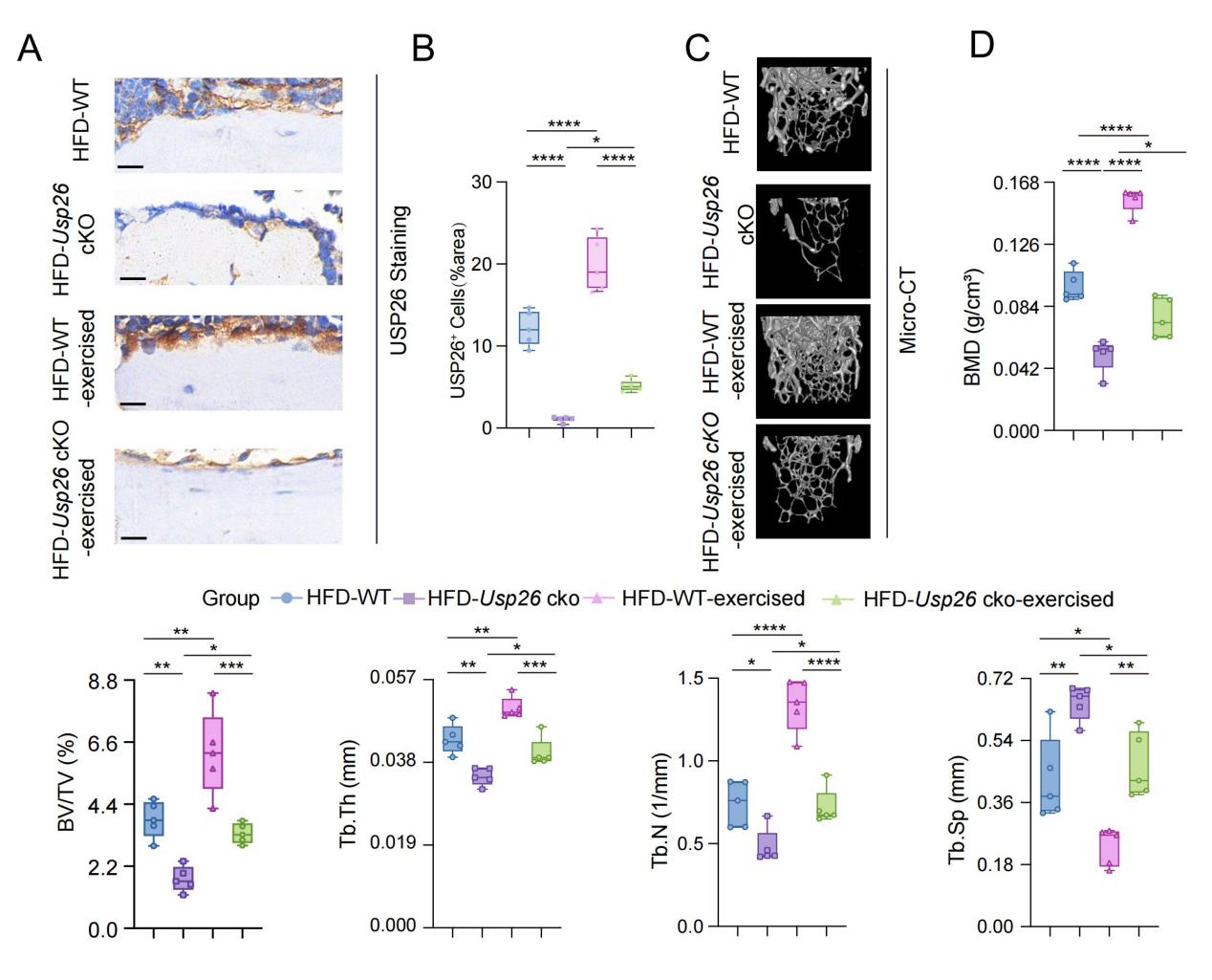


**Figure S10. Running exercise prevented the reduction of USP26 expression in osteoblasts and decreased bone mass in mice under high-fat diet conditions.** (**A, B**) Immunofluorescence staining of USP26 in femoral sections from Usp26 cKO mice and their littermate controls under high-fat diet followed by either exercise or not. n=5 in each group. Scale bars: 25μm. (**C, D**) Micro-CT reconstruction and quantitative analysis of trabecular bone in Usp26 cKO vs. littermate control mice under HFD with exercise or not. Statistical parameters: BV/TV (bone volume fraction), BMD (bone mineral density), Tb.N (trabecular number), Tb.Th (trabecular thickness), Tb.Sp (trabecular separation). n=5 in each group. Data are presented as mean ± SEM. Statistical significance was determined by one-way ANOVA. *p < 0.05, **p < 0.01, ***p < 0.001. ****p < 0.0001.


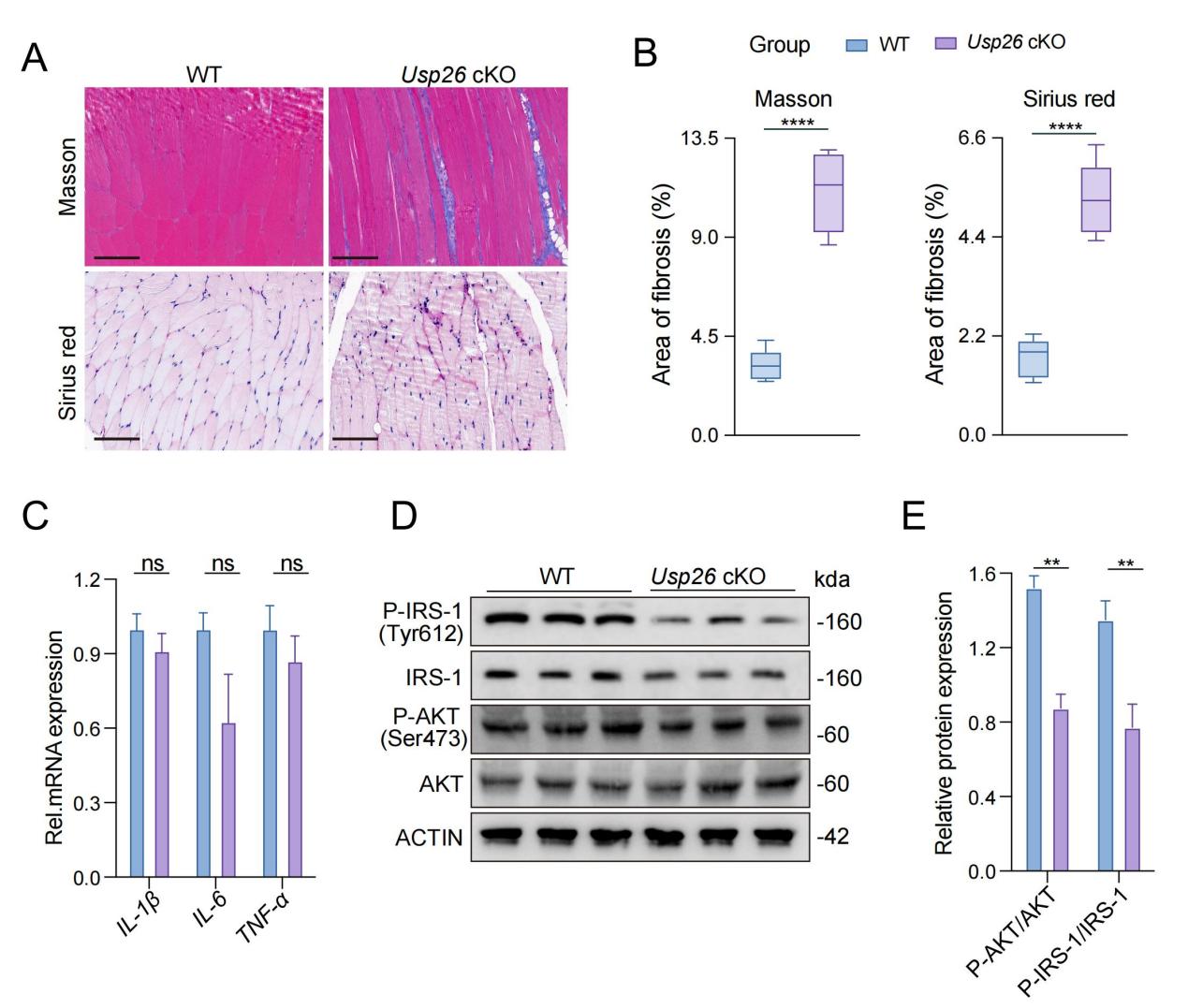


**Figure S11. Usp26 cKO mice exhibit significant skeletal muscle fibrosis and insulin resistance.** (**A**) Masson's trichrome staining and Sirius red staining of skeletal muscle tissues from Usp26 cKO mice and their littermate controls. n = 5 in each group. Scale bars: 100μm. (**B**) Statistical analysis of Masson's trichrome staining and Sirius red staining in (A). n=5 in each group. (**C**) qPCR analysis of IL-6, IL-1β, and TNF-α mRNA levels in skeletal muscle tissues from Usp26 cKO mice and littermate controls. n=3 in each group. (**D**) Western blot analysis of P-IRS1 (Tyr612) and P-AKT (Ser473) in skeletal muscle tissues from Usp26 cKO mice and littermate controls. n=3 in each group. (**E**) Statistical analysis of Western blot results in (D). n=3 in each group. Data are presented as mean ± SEM. Statistical significance was determined by two-tailed Student’s t-test. **p < 0.01, ****p < 0.0001.


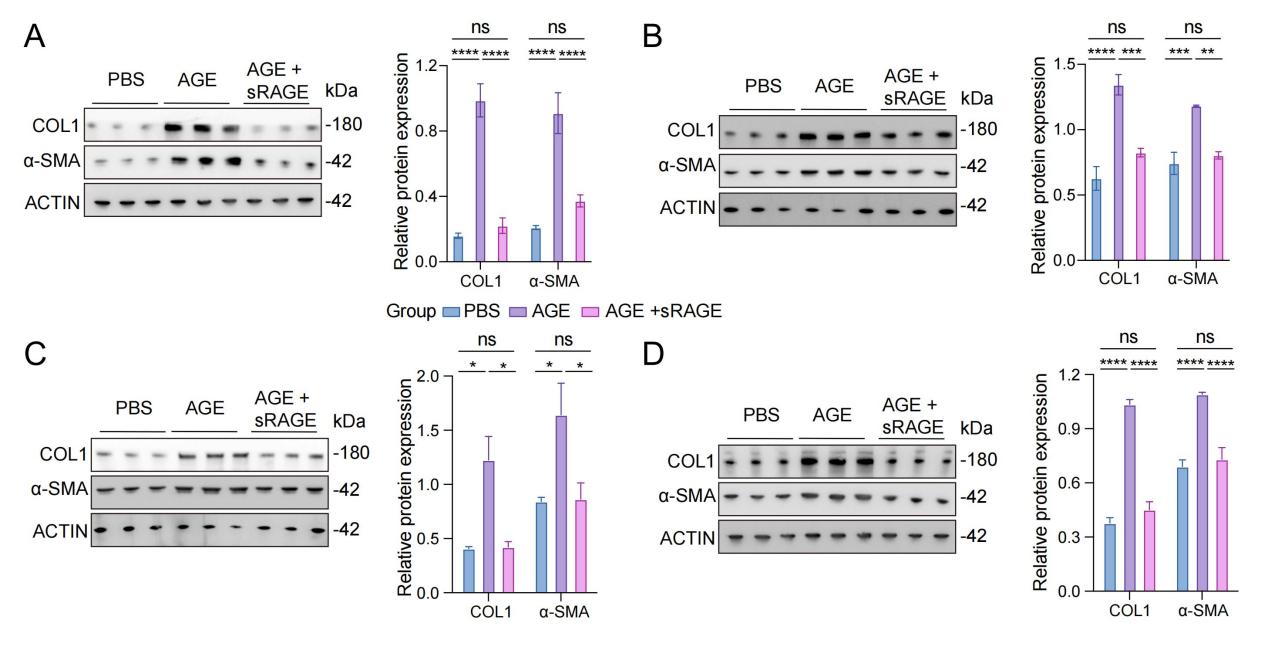


**Figure S12. The AGE-RAGE axis is critical for inducing myofibroblast differentiation.** (**A**) Hepatic stellate cells, (**B**) splenic fibroblastic reticular cells, (**C**) pulmonary fibroblasts, and (**D**) renal fibroblasts were treated with PBS, AGE, or AGE + sRAGE, respectively. Western blot was performed to detect the protein levels of α-SMA and COL1, followed by statistical analysis. n=3 in each group. Data are presented as mean ± SEM. Statistical significance was determined by one-way ANOVA. *p < 0.05, **p < 0.01, ***p < 0.001, ****p < 0.0001.


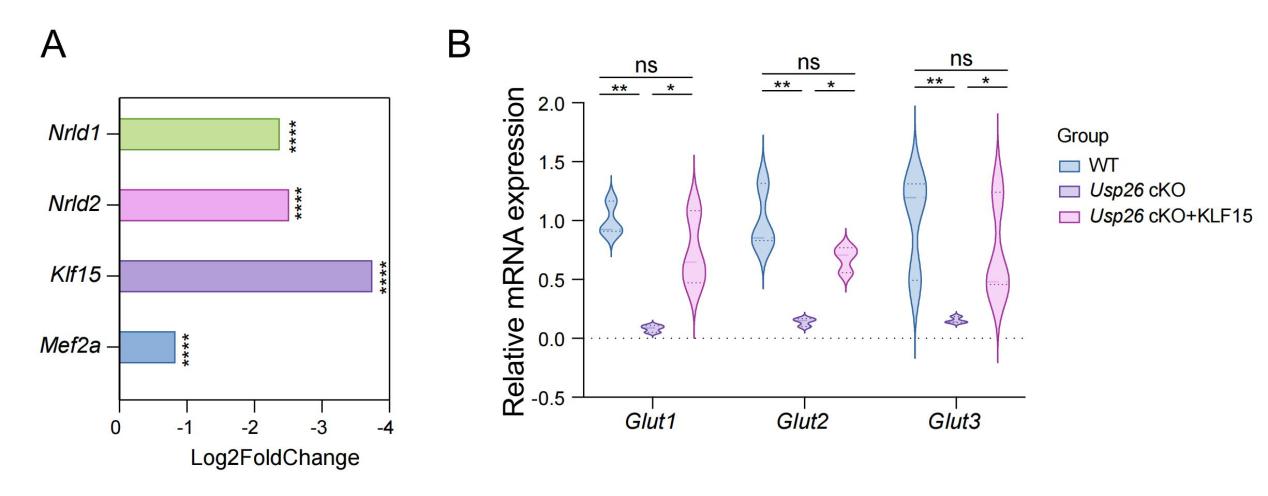


**Figure S13. Restoration of Klf15 expression rescues Glut1, Glut2, and Glut3 expression in Usp26 CKO osteoblasts.** (**A**) Differential expression analysis of Mef2a, Klf15, Nr1d2, and Nr1d1 in RNA-Seq data from osteoblasts of Usp26 cKO mice and their littermate controls, n=5 in each group. (**B**) qPCR analysis of GLUT1, GLUT2, and GLUT3 mRNA levels in osteoblasts from Usp26 cKO mice, littermate controls, and Usp26 cKO osteoblasts with KLF15 co-treatment, n=5 in each group. Data are presented as mean ± SEM. Statistical significance was analyzed using two-tailed Student’s t-test in (**A**) one-way ANOVA in (**B**), *p < 0.05, **p < 0.01, ****p < 0.0001.

**Table S1. Primer sequences for real time-PCR**

| **Gene** | **Forward primer** | **Reverse primer** | **Species** |
| --- | --- | --- | --- |
| Fstl1 | CACGGCGAGGAGGAACCTA | TCTTGCCATTACTGCCACACA | Mice |
| Ksrp | CTGTTTTGTTTGGCGAGAGAG | GAGACACAGAACAGGCGAGAG | Mice |
| Usp26 | TGGTGTGGATGTTCGTGATCT | CCACTCTAGGCCGTTCTCAATAC | Mice |
| Acca | AATGAACGTGCAATCCGATTTG | ACTCCACATTTGCGTAATTGTTG | Mice |
| Glut1 | GCAGTTCGGCTATAACACTGG | GCGGTGGTTCCATGTTTGATTG | Mice |
| Glut2 | ATCCCTTGGTTCATGGTTGCTG | TCCGCAATGTACTGGAAGCAG | Mice |
| Glut3 | TGGTAGCTCAGATCTTTGGTTTGG | GATCTCTGTAGCTTGGTCTTCCTC | Mice |
| Glut4 | CCAGCCACGTTGCATTGTA | ACACTGGTCCTAGCTGTATTCT | Mice |
| Pgk1 | ATGTCGCTTTCCAACAAGCTG | GCTCCATTGTCCAAGCAGAAT | Mice |
| Pdk1 | GGACTTCGGGTCAGTGAATGC | TCCTGAGAAGATTGTCGGGGA | Mice |
| Dha | TGTCTCCAGCAAAGACTACTGT | GACTGTACTTGACAATGTTGGGA | Mice |
| Glg1 | GAGCTGTGTAAAAAGGAAGTGTT | TTGAGGCGCTTTTTGCACTC | Mice |
| Alp | TCATTCCCACGTTTTCACATTC | GTTGTTGTGAGCGTAATCTACC | Mice |
| Bmp2 | AGTAGTTTCCAGCACCGAATTA | CACTAACCTGGTGTCCAATAGT | Mice |
| Runx2 | CCTTCAAGGTTGTAGCCCTC | GGAGTAGTTCTCATCATTCCCG | Mice |
| Osterix | ACCAGAAGCGACCACTTGAG | TTGGCTTCTTCTTCCCCGAC | Mice |
| Osteocalcin | GGTAGTGAACAGACTCCGGC | TTAAGCTCACACTGCTCCCG | Mice |
| β-actin | GGAGGGGGTTGAGGTGTT | GTGTGCACTTTTATTGGTCTCAA | Mice |
| Usp26 | GACCTGGTAAGGGTGGGAGT | TCTCCGCAAGTAAGTGTCAATTT | Human |
| β-actin | CACAGAGCCTCGCCTTTGC | AATCCTTCTGACCCATGCCC | Human |

**Table S2. Primer sequences for ChIP-qPCR**

| **Gene** | **Forward primer** | **Reverse primer** | **Species** |
| --- | --- | --- | --- |
| Ksrp | GGCCCAATGAAACGCGAGTGC | AGCACCACCTCTCACGTGCATTC | Mice |
